# Closing the perovskite loop: aqueous recovery and reuse of MAPbI_3_ for film, device, and nanocrystal reuse

**DOI:** 10.1039/d6mh01719c

**Published:** 2026-10-21

**Authors:** Zhaoyi Du, Ding Ding, Longren Li, Weitao Qian, Enny Tran, Milo S. P. Shaffer, Artem A. Bakulin, Nicola Gasparini, Martyn A. McLachlan

**Affiliations:** a Department of Materials, Molecular Sciences Research Hub, Imperial College London London W12 0BZ UK; b Department of Chemistry, Molecular Sciences Research Hub, Imperial College London London W12 0BZ UK martyn.mclachlan@imperial.ac.uk

## Abstract

The rapid commercialisation of organic–inorganic metal halide perovskites (MHPs) presents significant end-of-life challenges, particularly owing to the environmental and health risks their lead content poses. Despite efforts to improve perovskite device performance, comparatively little attention has been given to safe and sustainable recycling, with reported approaches being reliant on hazardous solvents, including DMF. Here, we report the development and use of a water-based solvent for the selective recovery of methylammonium lead iodide (MAPbI_3_) from MHP devices. The solvent comprises of water with low concentrations of dissolved malic acid, β-alanine, and sodium iodide (NaI), in which the organic additives enable MAPbI_3_ dissolution and NaI supports direct MHP recovery. Owing to the temperature dependent solubility of MAPbI_3_ in our solvent, high-purity powders can be recovered through precipitation by solvent cooling. Films fabricated from recycled MAPbI_3_ exhibit microstructures and bandgaps comparable to those prepared from pristine MAPbI_3_, whilst recycled films show improved morphological uniformity and reduced non-radiative recombination. In photoconductor devices, recycled MAPbI_3_ films deliver a higher photocurrent density, 1.48 × 10^−5^ A cm^−2^, and lower dark current density, 1.25 × 10^−7^ A cm^−2^ compared to their pristine analogues. When incorporated into p–i–n photovoltaic devices, recycled MAPbI_3_ films retain >90% of the efficiency of pristine devices. Furthermore, demonstrating the versatility of the solvent system beyond thin-film reuse, MAPbI_3_ recovered from thin films is successfully repurposed for the preparation of perovskite nanocrystals. Overall, this work establishes a green and efficient route for the recovery, recycling, and reuse of metal halide perovskites (MHPs), supporting a more circular lifecycle for emerging perovskite optoelectronics.

## Abstract 2

An aqueous solvent enables temperature-controlled recovery of MAPbI_3_, regenerating high-quality semiconductor materials for thin-film optoelectronic devices and nanocrystals, advancing circular perovskite technologies.

## Graphical abstract

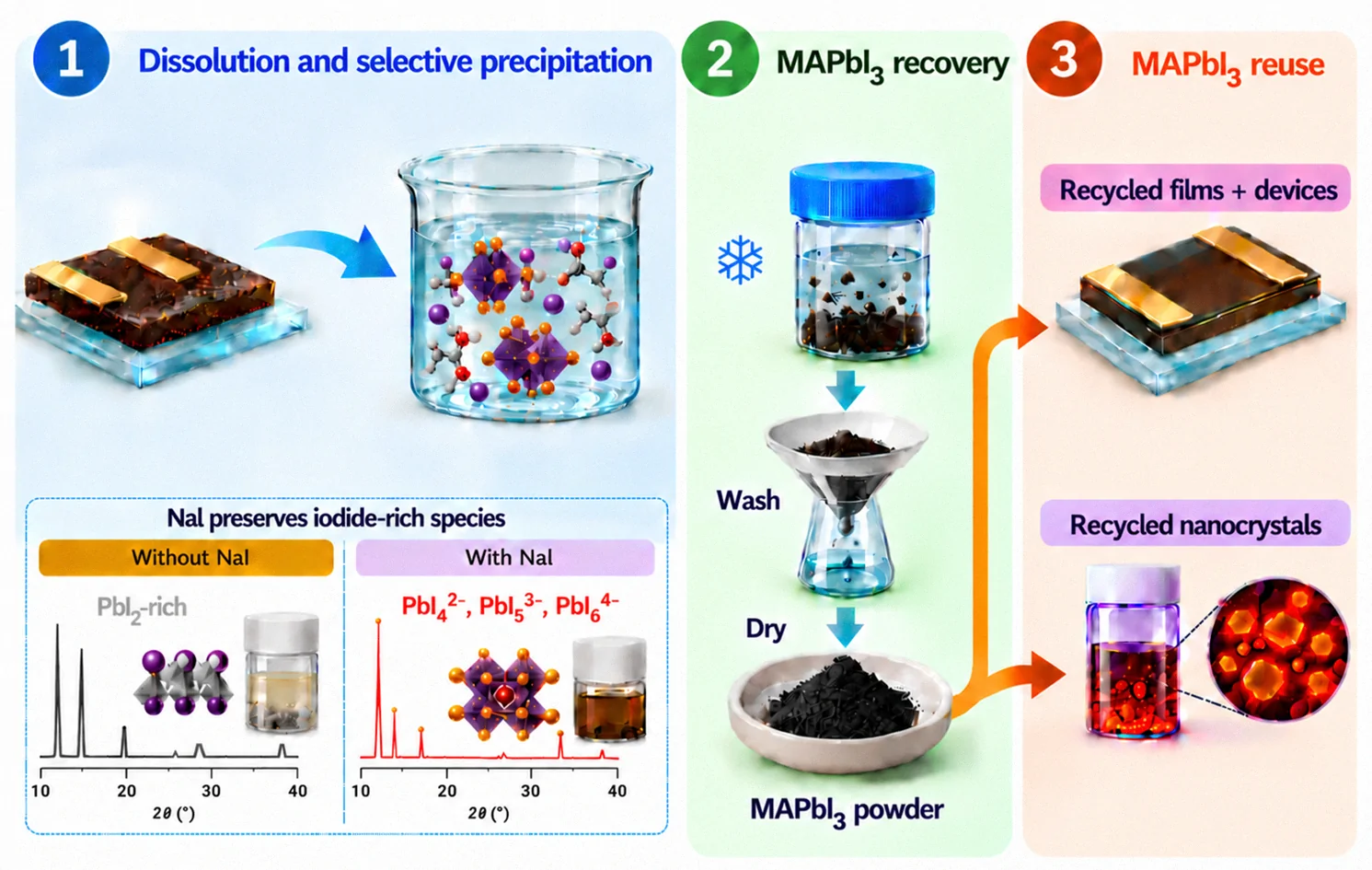

**New concepts**

Closing the perovskite loop: aqueous recovery and reuse of MAPbI_3_ for film, device, and nanocrystal reuse.

This work introduces a fully aqueous, green solvent system for the selective recovery, recycling, and functional reuse of methylammonium lead iodide (MAPbI_3_) from metal halide perovskite thin-film devices – establishing a closed-loop recycling strategy that avoids the hazardous organic solvents that underpin virtually all existing approaches. While prior recycling efforts have demonstrated lead capture or component recovery, none have achieved a complete loop in which recovered material is reprocessed into devices that surpass the performance of their pristine counterparts. This counterintuitive outcome combined with superior morphological uniformity, reduced non-radiative recombination, and enhanced photoconductor metrics in recycled devices arises from pre-formed perovskite species in solution that promote more controlled crystallisation, a mechanistic insight with broader implications for perovskite film deposition strategies. The additional demonstration that recovered MAPbI_3_ can be repurposed into luminescent nanocrystals further establishes the versatility and circularity of the platform. This work reframes recycling not as a remediation step, but as a route to functional enhancement, offering the materials community a sustainable, scalable, and low-cost pathway toward truly circular perovskite optoelectronics.

## 1. Introduction

Metal halide perovskites (MHPs) have emerged as a versatile class of semiconductors with broad potential spanning diverse optoelectronic applications. The rapid development of MHPs has been driven largely by their promise in photovoltaics (PVs), light-emitting diodes (LEDs) and photodetectors (PDs).^1–3^ Increasingly, their attractive photophysical and electronic properties are also being exploited in more diverse technologies, including lasers, sensors, and photocatalytic systems.^4–6^ In the PV sector there has been rapid progress towards commercialisation,^7–9^ with imaging, display and PD technologies rapidly nearing commercialisation.^10–12^ The expansion of research activity and commercial interest in MHPs has made the development of effective end-of-life capture and recycling strategies a priority. This is critical from the perspective of resource efficiency and broader sustainability and owing to the presence of lead. In humans, lead can adversely affect the central nervous, reproductive, developmental, cardiovascular and immune systems and when released is environmentally persistent.^13,14^ Innovative, highly effective recycling strategies have evolved whereby layer-by-layer dissolution of PVs have been developed,^15^ although current recycling strategies often depend on organic solvents for dissolution.^16^ Such solvents are critical for the deposition of MHP films and in post-deposition defect reduction.^17,18^ However, they also carry inherent toxicity and environmental concerns.^19,20^ This further refines the requirements for emerging capture and recycling strategies, emphasising that consideration must extend beyond recovery performance to the nature of the materials used in the process and the risks they may pose to human health and the environment. Remarkably this is an area that remains relatively unexplored, despite growing interest in end-of-life management of MHP materials and devices. Early attention focussed on the recycling of the hazardous and high-value PV components, with substrate/transparent conducting oxide recovery achieved through a multi-step lead capture process and subsequent reuse.^20^ More recently aqueous-based recycling strategies have begun to emerge, whereby multiple functional components are combined holistically to recover and regenerate MHP PVs. Taken together, these studies highlight both the promise of aqueous recycling and the fact that it remains underdeveloped relative to the broader maturity and scale of perovskite device research.

Recently we demonstrated an aqueous solvent system capable of supporting the size-controlled synthesis of MHP nanocrystals, yielding materials with excellent photon-to-electron conversion in simple photoconductor architectures.^21^ The solvent comprises water with low concentrations of dissolved malic acid and β-alanine where the synergistic interaction between malic acid and β-alanine promotes adduct formation with PbBr_2_, overcoming its otherwise poor aqueous solubility and enabling subsequent precipitation of the desired MHP phase. Here, we demonstrate the recovery and reuse of the MHP active layer using an optimised aqueous solvent system. Our aqueous solvent system dissolves the metal halide perovskite (MHP) layer, enabling direct capture and recovery of α-phase MAPbI_3_ in its original perovskite form – rather than as a decomposed precursor. This represents a key distinction from many existing perovskite recycling approaches, which typically recover only the lead halide salt (PbX_2_). Those that have demonstrated perovskite recovery do so use a series of organic solvents.^22^ In very recent work MAPbI_3_ recovery has been demonstrated using an acidic solution with additives that afford control of solubility, phase purity and stability.^23^ Table S1 benchmarks our approach against prior reports, comparing perovskite composition, solvent system, dissolution/recovery strategy, recovery efficiency, and post-recycling device performance.

Our recovered MAPbI_3_ is reprocessed *via* conventional routes to produce perovskite nanocrystals (PNCs) and thin films that, upon integration into photoconductor and PV devices, deliver performance metrics comparable to their pristine analogues. We further demonstrate the versatility of the process by converting recovered MHP films into emissive PNCs. Our aqueous solvent system provides an effective route for MHP recovery and subsequent reuse, enabling repurpose as functional thin films or luminescent nanocrystals.

## 2. Results and discussion

### Structural and morphological characterisation of dissolution and reprecipitation

Process optimisation was carried out using films MAPbI_3_, 400 nm thickness, deposited onto glass substrates in a one-step deposition (see methods). In contact with water, MAPbI_3_ typically undergoes hydration followed by irreversible degradation, forming soluble methylammonium and iodide species and insoluble PbI_2_ (Fig. S1). A distinct visual modification of the substrate is observed as the film dissolves in our aqueous solution composed of malic acid, β-alanine and H_2_O, changing from the characteristic black colour of MAPbI_3_ to the transparent appearance of glass (Fig. S1). No precipitation is observed, the insoluble PbI_2_ is solubilised through adduct formation with malic acid, confirmed using UV-vis absorption spectroscopy shown in Fig. S2. The spectra show an absorption peak centred at 293 nm, attributed to PbI_2_(Sol)_4_ iodoplumbates (where Sol is the aqueous solvent), confirming that molecular PbI_2_ remains.^24^ There is a small and broad absorption centred at 360 nm that is attributed to PbI_4_^2−^.^25^

β-alanine deprotonates the malic acid forming carboxylate groups that, in-turn interact with the insoluble PbI_2_ to form a soluble adduct, Fig. 1a. ^1^H NMR was conducted on dl-malic acid and on a dl-malic acid/β-alanine mixture, using D_2_O as the solvent (Fig. S3). The methine proton (–C**H̲**(OH)–) peak of pure dl-malic acid centred at 4.56 ppm, shifting upfield to 4.33 ppm following addition of β-alanine. This upfield shift reflects increased electron shielding, consistent with the formation of carboxylate groups and thus deprotonation of malic acid. To further verify that the dissolution of PbI_2_ is facilitated by protonated carboxylate groups in malic acid, we replaced β-alanine with NaOH (p*K*_b_ values >3 and <1 respectively). Again, the absorption spectra confirm the presence of PbI_2_(Sol)_4_ iodoplumbates with the lower energy peak not observed, Fig. S4, attributed to the strong base favouring complete deprotonation of the malic acid and the formation of a single product, PbI_2_(Sol)_4_.

**Fig. 1:**
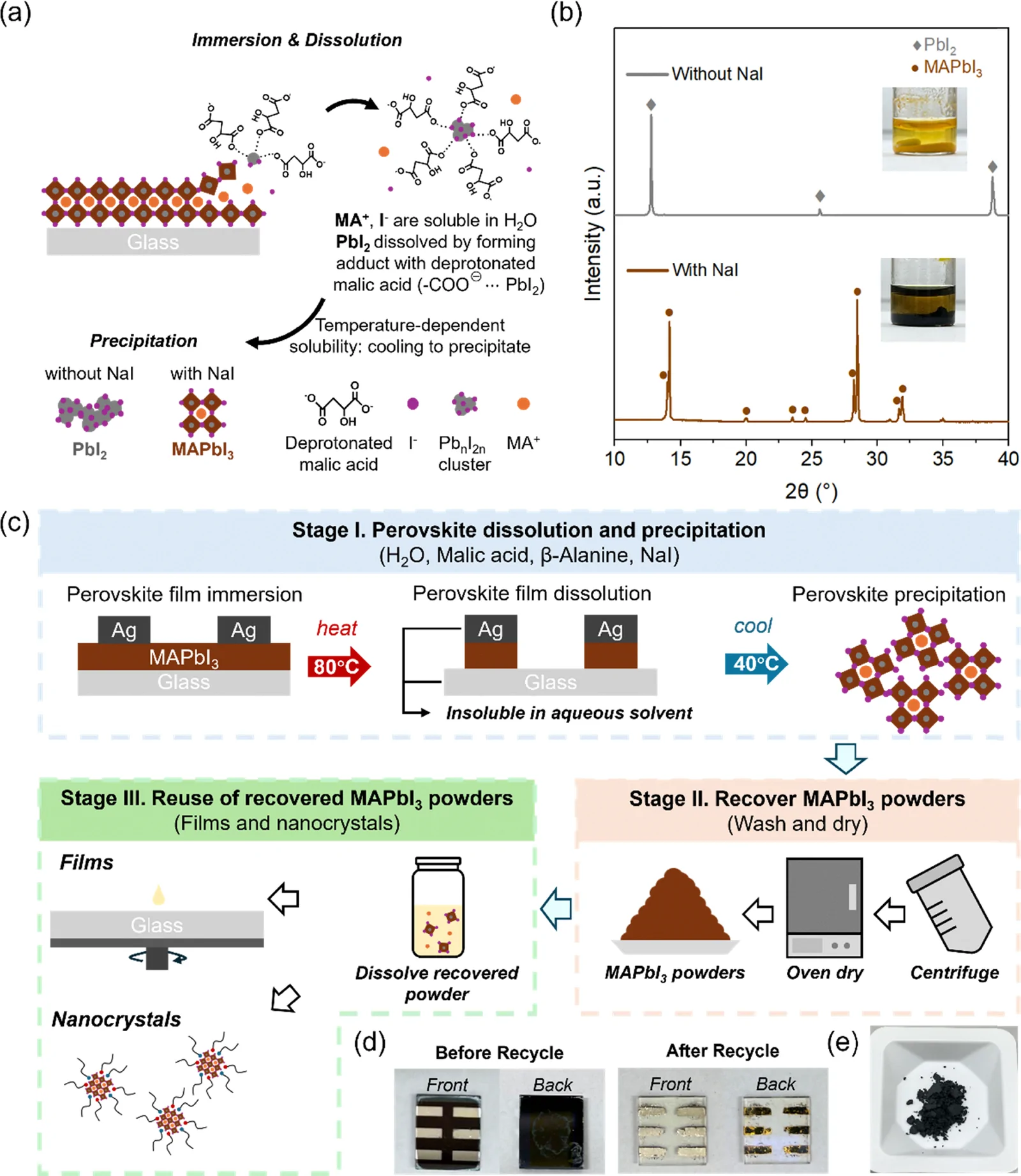
(a) Schematic illustration of MAPbI_3_ film dissolution and subsequent precipitation using the aqueous solvent system. (b) X-ray diffraction patterns of the solids recovered in the presence and absence of NaI – inset photographs show the corresponding precipitates in their respective solutions. (c) Schematic overview of the complete MAPbI_3_ recovery, recycling and reuse process. (d) Photographs of photoconductor devices before and after removal and recovery of the MAPbI_3_ layer. (e) Photograph of the recovered and dried MAPbI_3_ powder.

The solubility of MAPbI_3_ in the aqueous solvent is highly temperature dependent, Fig. S5, making cooling a convenient route to induce precipitation. In the absence of NaI, cooling produced a yellow precipitate and yellow suspension, consistent with PbI_2_ formation, Fig. 1b. The addition of NaI to the solvent had no discernible effect on the dissolution process but significantly altered the nature of the solid recovered upon cooling. The precipitates were isolated by centrifugation, the supernatant removed, and the solids were washed with HI/IPA (see methods) before drying at 60 °C overnight in ambient air, following which X-ray diffraction (XRD) was performed, Fig. 1b. The yellow powder recovered was confirmed as PbI_2_, with characteristic reflections at 12.77°, 25.59°, and 38.76° 2*θ*, assigned to the (001), (002), and (003) planes of hexagonal PbI_2_ respectively.^26^ In contrast, the powder recovered from the solvent containing NaI exhibited a diffraction pattern characteristic of MAPbI_3_. Reflections at 14.02° and 14.15° 2*θ* are assigned to the (002) and (110) planes of tetragonal MAPbI_3_ with the *I*4/*mcm* space group,^27^ additional peaks at 28.17° and 28.48° 2*θ* correspond to the (004) and (220) planes respectively (ICSD 01-085-5507). These results demonstrate that the aqueous solvent system dissolves MAPbI_3_ from the substrate and that NaI addition enables recovery of MAPbI_3_, rather than decomposition to PbI_2_.

To determine the role of NaI, UV-vis measurements were carried out on aqueous solvent systems containing: (i) MAPbI_3_ without NaI, (ii) MAPbI_3_ with NaI, and (iii) NaI without MAPbI_3_, as shown in Fig. S6. Distinct differences are seen in the spectra in the 340–380 nm range, solution (i) shows no peaks in this range, whereas solutions (ii) and (iii) show peaks 370 and 358 nm respectively. The peak in solution (i) is attributed to the presence of PbI_3_^−^,^28^ anticipated in iodide-rich conditions where PbI_2_ can form a range iodoplumbates, *e.g.* PbI_3_^−^, PbI_4_^2−^.^29,30^ Solution (iii) shows an absorption peak at 358 nm, originating from I_3_^−^.^31^ The additional iodide anions present in the solutions containing NaI drive the formation of lead species with increased iodide coordination *i.e.* PbI_4_^2−^, PbI_5_^3−^, PbI_6_^4−^ putting the lead into an octahedral coordination that readily results in the formation of MAPbI_3_.^32^

ATR-FTIR and Raman spectroscopy were conducted to investigate further the dissolution/reprecipitation mechanism and the role of NaI. In the ATR-FTIR spectra, the band at 1710 cm^−1^, assigned to *v*(C=O) of –COOH groups, decreases in intensity upon dissolution of MAPbI_3_ and addition of NaI. The characteristic asymmetric and symmetric COO^−^ stretching bands, located at 1630 and 1385 cm^−1^ in the aqueous solvent, shift to 1626 and 1378 cm^−1^ after MAPbI_3_ dissolution, indicating interactions between MAPbI_3_ and the organic molecules. Upon addition of NaI, the asymmetric COO^−^ band shifts markedly to lower wavenumber, overlapping with adjacent bands to form a broad feature centred at 1573 cm^−1^. This pronounced shift is consistent with the reported formation of Pb–carboxylate interactions.^33^ Raman spectroscopy was conducted on the MAPbI_3_ film and on MAPbI_3_ dissolved in our solvent with and without NaI (Fig. S8), the peak positions given in Table S2. The low-wavenumber region (50–150 cm^−1^) is assigned to vibrations of the Pb–I cage in MAPbI_3_ and PbI_2_, while the higher-wavenumber region (175–450 cm^−1^) is typically assigned to methylammonium-related vibrations in MAPbI_3_.^34^ The MAPbI_3_ film shows a series of peaks in the higher-wavenumber region that disappear following dissolution, indicating a loss of the the 3-D MAPbI_3_ structure. In the solvent without NaI, three sharp, well-resolved peaks are observed at 61.3, 86.5 and 106.1 cm^−1^*i.e.* in the low-wavenumber region, assigned to mixed I–Pb–I bending and Pb–I stretching modes, respectively.^35–37^ Such sharp, PbI_2_-like features have been attributed to perovskite decomposition.^38^ In the presence of NaI, these Pb–I peaks broaden and shift to higher wavenumber, an additional peak around 136.5 cm^−1^ also appears, assigned to the antisymmetric I–I stretch of distorted I_3_^−^.^39^ The broadening is consistent with a distribution of Pb–I coordination environments, rather than solely PbI_2_. The increased iodide concentration will favour the formation of iodide-rich iodoplumbate species, potentially including [[PbI_4_]^2−^ and [PbI_5_]^3−^,^39^ consistent with the UV-vis spectra, Fig. S6. Such species may preserve high Pb–I coordination during dissolution, thereby facilitating subsequent reconstruction of the PbI_6_ framework during MAPbI_3_ recovery.^34,39,40^

### Assessment of lead recovery

Having demonstrated the dissolution and re-precipitation of MAPbI_3_ in our aqueous solvent, we next assess the feasibility of recovering MAPbI_3_ directly from perovskite devices. For this, we use a simple photoconductor with the structure glass/MAPbI_3_/Ag, Fig. 1c. Devices were immersed in the aqueous solvent at 80 °C, resulting in dissolution of the black MAPbI_3_ film, exposing the underlying substrate while leaving the electrodes intact, Fig. 1d. The selectivity of the aqueous solvent toward other materials commonly encountered in perovskite devices was also investigated. Hole transport layers (NiO_*x*_) and electron transport layers (SnO_2_), deposited as films on ITO-coated glass, were immersed in the aqueous solvent for 3 minutes. Analysis, Fig. S9, confirms that neither is soluble in our solvent, indicating selectivity towards the perovskite layer. Subsequent cooling of the resulting solution <40 °C induced MAPbI_3_ precipitation, which was then separated, washed, and dried to yield MAPbI_3_ powder, Fig. 1e. Following removal of the MAPbI_3_ the solution was used to capture fresh MAPbI_3_, and this dissolve–precipitate–separate cycle was repeated over multiple rounds. After each round, recovery yield was determined as:
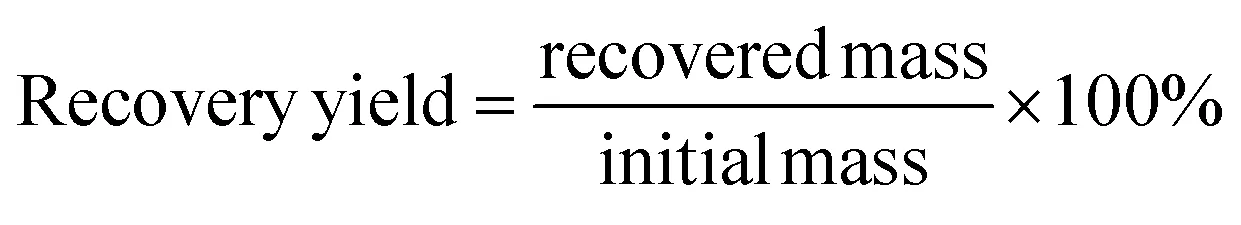


Fig. S10 compares the recovery yield obtained using freshly prepared aqueous solvent against solvent that had already been reused following precipitate removal. Notably, solubility never falls to zero, thus a portion of the Pb that is solubilised on the first solvent use is retained. On subsequent rounds of reuse the recovered quantity matches the difference in solubility shown in Fig. S10 where the recovery yield is >80%.

Building on the recovery yield evaluation, we further compared the toxicity of our aqueous solvent against organic solvents and water, Table S3. Organic solvents including DMF, DMSO, and GBL can cause serious health effects under prolonged exposure, whereas malic acid and β-alanine, the key components of our solvent, show no appreciable acute toxicity at the concentrations used here. NaI is present at a comparatively high concentration, as an iodine-rich environment is essential for stabilising the α-phase of MAPbI_3_ during recovery; while this raises some toxicity concern, it remains substantially lower than that of the organic solvents. Pure water, by contrast, is essentially non-hazardous, but its use alone yields PbI_2_, reducing the recovery efficiency.^41^

### Reuse of recovered MAPbI_3_

When the precipitate is removed the solvent can again be further reused, outlined schematically in Fig. 1c. The recovered MAPbI_3_ was subsequently dissolved in DMF: DMSO (see methods) and spin-coated to glass substrates creating recycled films. Their structure was compared with pristine films prepared by conventional one-step deposition using XRD, Fig. 2a. The diffraction patterns are largely similar, with peaks consistent with tetragonal MAPbI_3_ (ICSD 01-085-5507) with no discernible differences in peak position or relative intensity between the samples. A weak peak at approximately 12.6° 2*θ* is observed in the recycled film, indicating a small quantity of PbI_2_. Given the structural similarity between the films, their optical bandgaps were determined by UV-vis spectroscopy and corresponding Tauc analysis, Fig. 2b. The extracted bandgaps are within experimental error of one another, at approximately 1.61 eV, consistent with reported values for MAPbI_3_.^42^

**Fig. 2:**
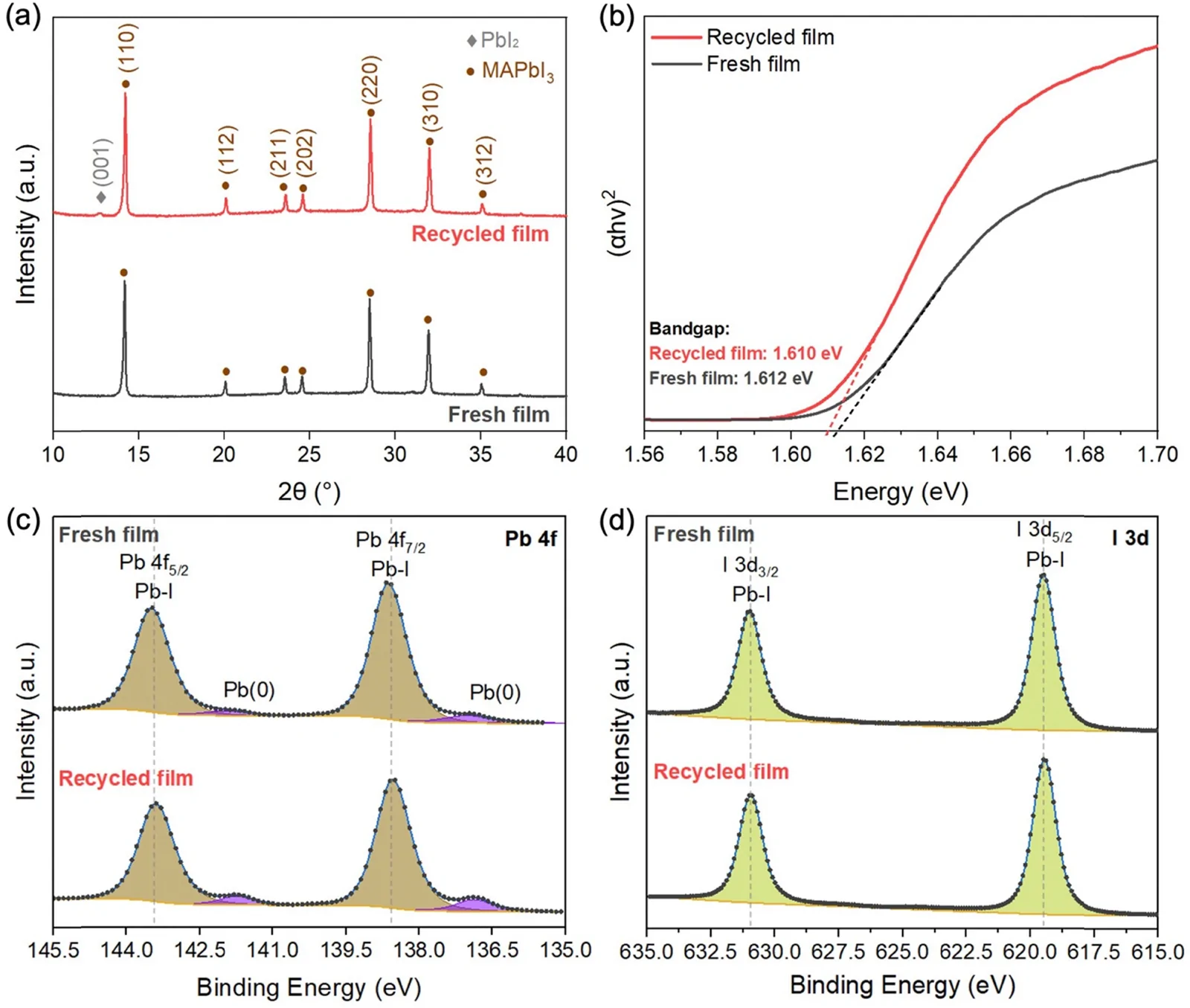
Comparison of the structural, optical and chemical properties of pristine and recycled MAPbI_3_ films: (a) X-ray diffraction patterns. (b) Tauc plots and high-resolution XPS spectra of the (c) Pb 4f and (d) I 3d regions.

The comparable structural and optical properties motivated further comparison of the film surface chemistry using X-ray photoelectron spectroscopy (XPS). Survey spectra, Fig. S11a, confirm the presence of Pb, I, N and C in both pristine and recycled films, with no additional elements detected following the dissolution-recrystallisation process. Closer inspection of the high-resolution C 1s spectra (Fig. S11b) reveals three components centred at 284.8, 286.3 and 287.6 eV, assigned to adventitious C–C/C–H, methylammonium carbon (with possible overlap from C–O species),^43,44^ and surface carbonyl (C=O) species, respectively. Compared with the pristine film, the overall fitted C–N/C–O component in the recycled sample exhibits a slight shift (∼0.2 eV) toward higher binding energy, accompanied by a small increase in relative intensity. This is attributed to an increase in the relative contribution of C–O with respect to C–N. As these two signals overlap significantly in the XPS spectra and given that C–O typically appears at a slightly higher binding energy than C–N, a greater C–O contribution naturally manifests as an apparent shift toward higher binding energy. The elevated C–O contribution likely originates from oxygen-containing functional groups present in the organic molecules, malic acid and/or β-alanine. The Pb 4f and I 3d spectra also show a high degree of similarity, Fig. 2c and d. For both films, the Pb 4f spectrum is dominated by the characteristic Pb^2+^ doublet, with the Pb 4f_7/2_ and Pb 4f_5/2_ peaks centred at 138.6 and 143.4 eV, respectively, while the I 3d_5/2_ and I 3d_3/2_ peaks are located at 619.4 and 630.9 eV, with all binding energies falling within the range of reported values for MAPbI_3_.^45–47^ These observations indicate that a Pb–I bonding environment comparable to that of pristine material is reestablished after recycling. In addition to the Pb^2+^ doublet, weak metallic Pb^0^ components are observed at approximately 136.9 eV and 141.8 eV in both the pristine and recycled films, arising from X-ray-induced reduction during measurement. As undercoordinated surface Pb is known to be particularly susceptible to such reduction,^48,49^ the comparatively higher relative intensity of the metallic Pb^0^ component suggests that the recovered MAPbI_3_ exhibits reduced MAI passivation at the surface, most likely a consequence of preferential MAI dissolution during the IPA washing step of the recycling process.^50^

The surface morphology and thickness of the fresh and recycled films were analysed using scanning electron microscopy (SEM), Fig. 3a. Both films show a similar, compact polycrystalline structure, anticipated for MHP films, and are of comparable thickness. Analysis of the grain size distribution, Fig. S12, shows that the recycled films have subtly increased grain sizes and a narrower distribution. This increase in grain size and improved film uniformity may arise from the pre-formed ABX_3_ perovskite structure of the recycled material. Following dissolution in DMF/DMSO and subsequent deposition, these pre-existing perovskite species likely alter the crystallisation pathway relative to fresh precursor solutions, promoting the formation of more compact and uniform films.^51^

**Fig. 3:**
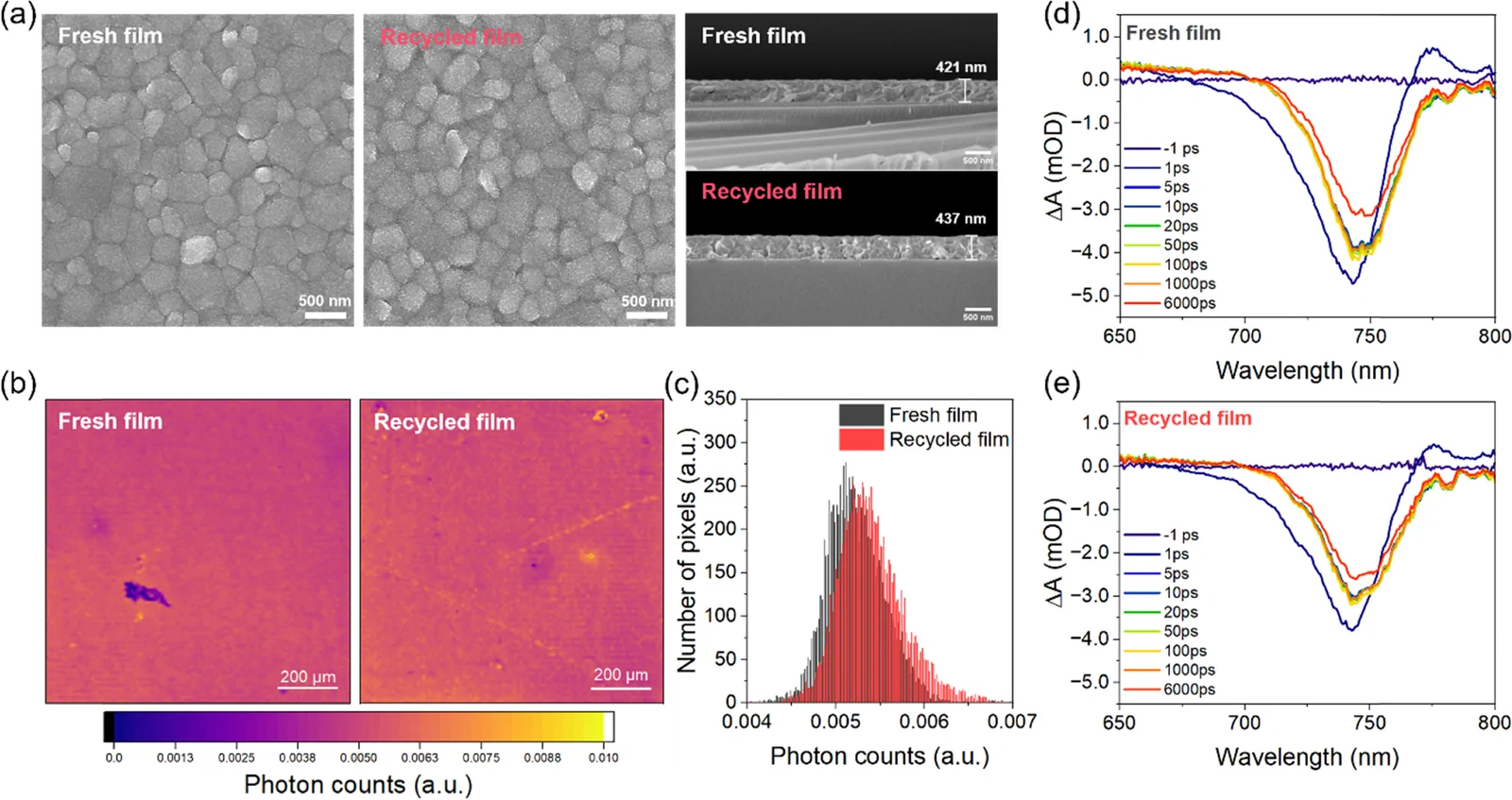
Comparison of fresh and recycled MAPbI_3_ films and photoconductor devices. (a) Top-view and cross-sectional SEM images. (b) Spatially resolved photoluminescence (PL) intensity maps and (c) corresponding photon-count distributions. Transient absorption (TA) spectra of (d) fresh and (e) recycled MAPbI_3_ films at selected pump–probe delays following 400 nm excitation at a pump fluence of 0.4 µJ cm^−2^.

### Spectroscopic characterisation

Steady-state photoluminescence (PL) spectra of fresh and recycled films showed peaks at 772 nm and 770 nm respectively, Fig. S13. Spatially resolved PL mapping was also carried out on fresh and recycled films, Fig. 3b. Both show largely homogeneous PL intensity distributions across the mapped areas, indicating good macroscopic film uniformity. Under identical excitation and acquisition conditions, the recycled film shows a slightly higher mean PL intensity than the fresh film, Fig. 3c. This enhanced PL response is consistent with reduced non-radiative recombination, which is commonly associated with trap-mediated losses at surfaces and grain boundaries.^52^ The spatially uniform PL response of the recycled film is also consistent with its compact morphology observed by SEM, Fig. 3a, and its narrower grain-size distribution, Fig. S12, together supporting improved microstructural uniformity.^53^

To assess whether the recycling process alters the photoexcited carrier dynamics of MAPbI_3_, we performed femtosecond transient absorption (TA) spectroscopy on fresh and recycled films deposited on glass substrates, Fig. 3d and e. Upon photoexcitation, both samples display a pronounced negative band-edge bleach at ∼730–750 nm, primarily attributed to photoinduced state filling, together with weaker broadband photoinduced absorption (PIA) features across 550–700 nm. Crucially, the fresh and recycled films exhibit highly similar spectral profiles and temporal evolution. No new transient features or shifts in the band-edge bleach are observed after recycling. Taken together, these observations suggest that the recycling process preserves the band-edge electronic structure and dominant relaxation pathways of MAPbI_3_.

To further assess the carrier dynamics, we analysed the normalised band-edge bleach kinetics extracted at 750 nm, Fig. S14. Two distinct temporal regimes are evident. The initial sub-picosecond evolution is associated with rapid carrier thermalisation and cooling towards the band edge, together with the establishment of the band-edge carrier population. As these early-time processes are sensitive to local energetic disorder and local defects, the closely matched sub-picosecond dynamics suggest that recycling does not appreciably alter the local carrier-relaxation environment. This is followed by a much slower decay extending into the nanosecond regime, reflecting carrier recombination influenced by long-lived defect states. The long-time dynamics are therefore sensitive to recombination-active trap states, particularly deep traps that contribute to long-lived carrier decay. The close agreement between the fresh and recycled films across both temporal regimes indicates that the dominant carrier-cooling and recombination pathways remain largely unchanged after recycling.

Time-correlated single photon counting (TCSPC) measurements under 467 nm excitation were performed to provide complementary information on the carrier dynamics (Fig. S15). The biexponential fitting parameters are summarized in Table S4. The recycled perovskite film presents comparable lifetime (18.7 ns) to fresh perovskite films (16.8 ns). The modest increase in lifetime observed in the recycled film originates predominantly from a redistribution of the decay-component amplitudes rather than a systematic increase in the individual time constants. Specifically, the relative amplitude of the long-lived component increases from 0.22 to 0.28, whilst that of the short-lived component decreases from 0.78 to 0.72, the corresponding time constants remain comparable between the two samples. Together with the film morphology and PL mapping results, this modest increase in PL lifetime is consistent with reduced non-radiative recombination in the recycled film, The TA measurements indicate that recycling preserves the ultrafast-to-early-nanosecond carrier dynamics without introducing additional defect-mediated recombination.

### Preparing devices from recovered MAPbI_3_

To compare the optoelectronic properties of films fabricated from recycled MAPbI_3_ with those prepared from pristine MAPbI_3_, we initially prepared simple photoconductor devices (Fig. 4a), enabling a direct comparison of bias-dependent dark and illuminated current density-voltage measurements.

**Fig. 4:**
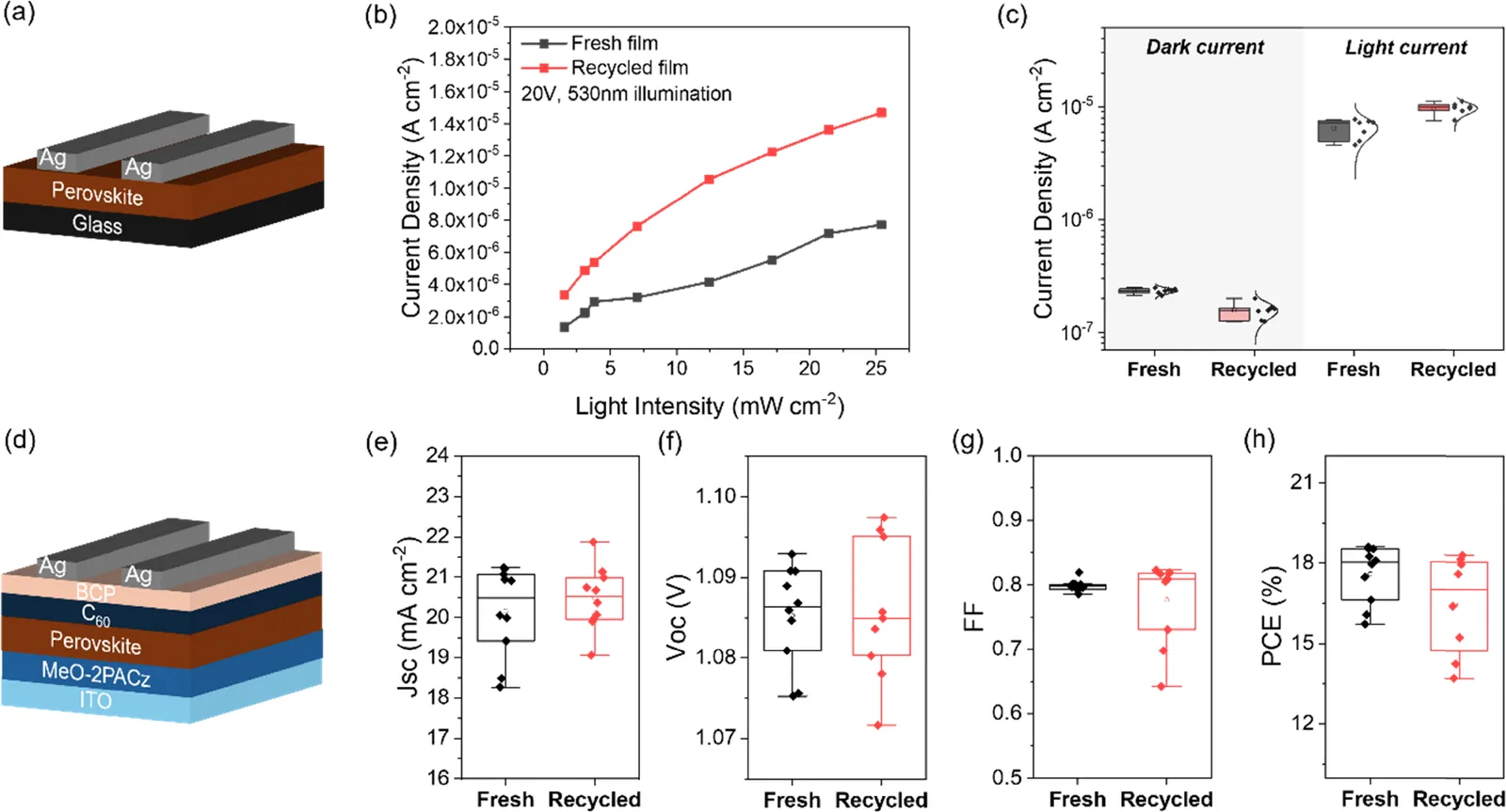
(a) Schematic illustration of the photoconductor device structure. Photoconductor devices fabricated using fresh and recycled MAPbI_3_ were characterised under 530 nm illumination. (b) Current density as a function of illumination intensity at an applied bias of 20 V, and (c) statistical comparison of the light and dark current densities, with the illuminated measurements obtained at 25.4 mW cm^−2^. (d) Schematic illustration of the photovoltaic device structure. Statistical distributions of the key photovoltaic performance parameters for devices prepared using fresh and recycled MAPbI_3_, presented as box-and-whisker plots: ((e) short-circuit current density (*J*_sc_), (f) open-circuit voltage (*V*_OC_), (g) fill factor (FF) and (h) power conversion efficiency (PCE).

The devices were illuminated using a 530 nm light source under varying illumination power and applied voltage, Fig. S16. Both sets of devices show a clear, monotonic increase in measured current density with increasing illumination intensity. The photocurrent density shows a strong dependence on light power. Interestingly the recycled films generate comparable current densities than the pristine counterparts at all illumination intensities. To visualize this difference more clearly, both types of devices were biased at 20 V and illuminated with an illumination intensity ranging from 2–25 mW cm^−2^, while the corresponding current densities were recorded, Fig. 4b. The current density of the device prepared from the pristine MAPbI_3_ increased from 0.13 × 10^−5^ to 0.7 × 10^−5^ A cm^−2^, whereas the recycled device increased from 0.35 × 10^−5^ to 1.48 × 10^−5^ A cm^−2^, suggesting a reduced defect density in the recycled film, consistent with the PL results. Subsequently, at 20 V, the dark current and light current densities of both devices were measured, Fig. 4c. The recycled films exhibit a dark current density (1.5 × 10^−7^ A cm^−2^) that is slightly lower than the pristine film (2.4 × 10^−7^ A cm^−2^). Overall devices prepared using recycled MAPbI_3_ have comparable, arguably improved light-to-current performance than devices prepared from pristine MAPbI_3_.

While photoconductors provide an excellent platform for evaluating the intrinsic optoelectronic properties of the recycled perovskite, the practical relevance of a recycling methodology ultimately depends on its compatibility with complete, multi-layer device architectures, in which multiple functional interfaces critically govern overall performance. To address this, full PV devices incorporating recycled MAPbI_3_ were fabricated, Fig. 4d and benchmarked against pristine counterparts, Fig. 4e–h. The devices employed a p–i–n structure, comprising a solution-processed MeO-2PACz hole transport layer and a thermally evaporated C_60_/BCP electron transport layer. The average PCEs of the pristine and recycled PSCs were 17.5 and 16.5%, respectively, with the recycled devices retaining 94% of the pristine PCE. This variation is primarily attributable to differences in fill factor, which is closely linked to interfacial quality. Notably, the box chart of PCE values for the recycled PSCs shows that some devices achieved efficiencies of up to 18%, comparable to the pristine counterparts.

### Synthesis of perovskite nanocrystals using recovered MAPbI_3_

To demonstrate the broader versatility of the solvent system, the MAPbI_3_ recovered from thin films was successfully reused for the synthesis of perovskite nanocrystals (PNCs), extending the value of the recycled material beyond the original application. Perovskite thin films have had tremendous impact in light harvesting applications whereas PNCs have been utilised in light-emitting applications such as LEDs, Fig. 5a. Briefly, the recycled MAPbI_3_ powder was dissolved in a solution of DMF containing oleic acid (OA) and oleylamine (OAm) at room temperature with vigorous stirring. This solution was then injected into chloroform resulting in the formation of PNCs, indicated by the red emission of the solution under 365 nm UV illumination, Fig. 5b.

**Fig. 5:**
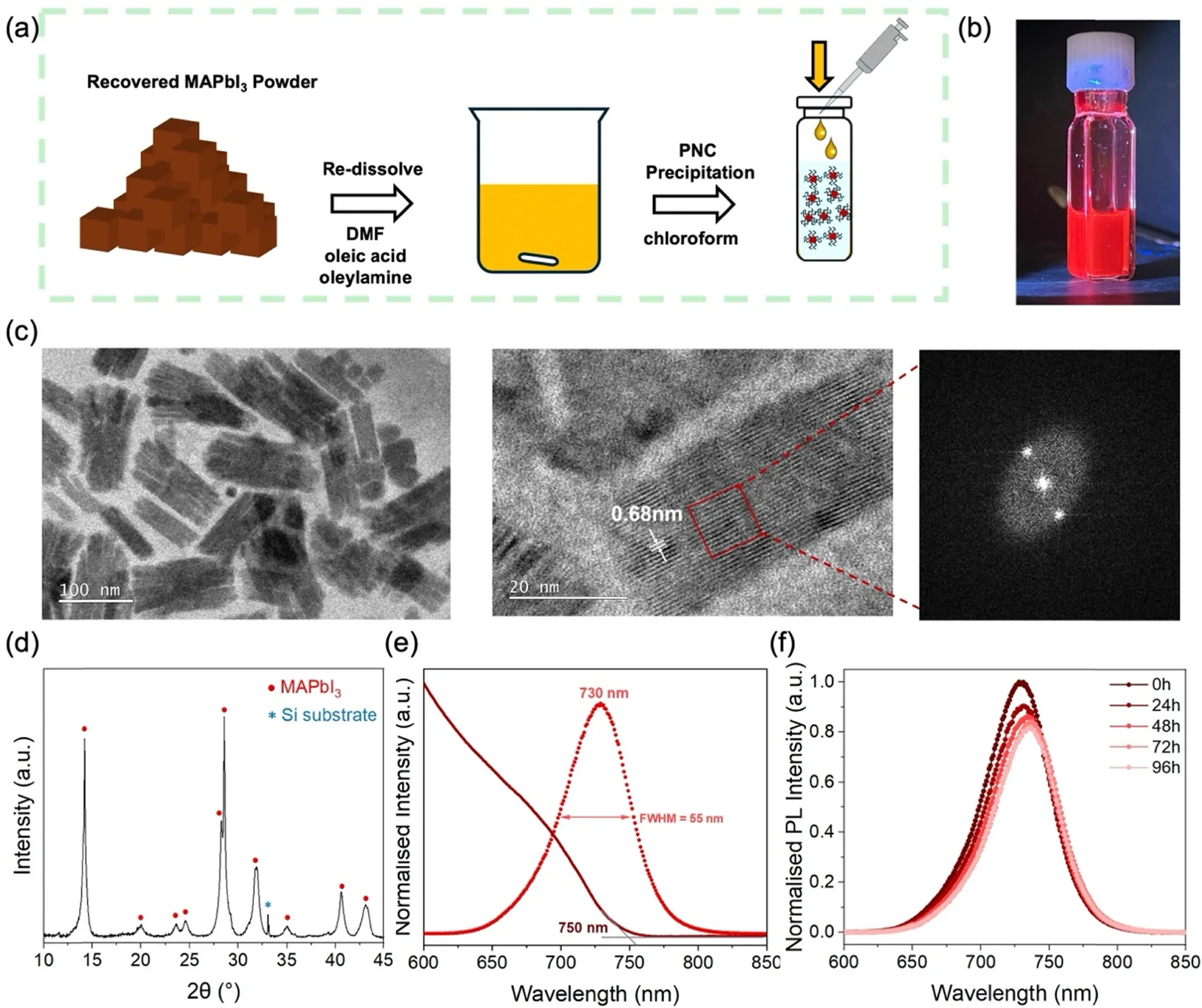
Synthesis and characterisation of MAPbI_3_ perovskite nanocrystals (PNCs) derived from recycled MAPbI_3_ powder. (a) Schematic illustration of the PNC synthesis process. (b) Photograph of the resulting PNC dispersion under UV illumination. (c) TEM and HRTEM images of the PNCs with the corresponding electron diffraction pattern. (d) X-ray diffraction pattern of the MAPbI_3_ PNCs. (e) UV-visible absorption and photoluminescence (PL) spectra of the PNC dispersion. (f) Normalised PL spectra recorded as a function of storage time.

TEM analysis revealed rod-shaped PNCs with average dimensions of approximately 18 nm along the short axis and 100 nm along the long axis, Fig. 5c. High-resolution TEM (HR-TEM) confirmed crystallinity, showing well-defined lattice fringes consistent with the (110) planes of tetragonal MAPbI_3_.^54^ This was further confirmed by XRD, which showed the tetragonal MAPbI_3_ phase with the *I*4/*mcm* space group. Diffraction peaks at 14.2° and 28.5° 2*θ* correspond to the tetragonal (110) and (220) planes respectively, Fig. 5d. PNCs an additional resolved peak at 28.2° 2*θ* is seen, assigned to the (004) plane, indicating a different preferred crystal-growth orientation in the PNCs.^55^ The full width at half maximum (FWHM) of the (110) peak at 14.23° also increased from 0.13° 2*θ* for the film to 0.22° 2*θ* for PNCs, consistent with a reduction in crystallite size.

The optical properties and stability of the PNCs were assessed by UV-vis absorption and PL spectroscopy. The PNC dispersion exhibited an absorption onset at approximately 750 nm and a PL emission maximum at 732 nm, with a FWHM of 55 nm, Fig. 5e. To evaluate colloidal stability, the PNCs were dispersed in chloroform, stored under ambient conditions, and their PL spectra recorded at 24 hour intervals, the integrated PL intensity being normalised to that of the freshly prepared dispersion. After 96 hours, the PNCs retained 81% of their initial PL intensity, indicating reasonable short-term stability, Fig. 5f. A progressive red-shift in the PL maximum was also observed, which is associated with the labile ligands, OA and OAm, that bind to the PNC surfaces dynamically.^56^ Ligands desorption occurred during aging, leading to trap state formation and aggregation that produces a PL red-shift.^57,58^

The dimensions and optical properties of perovskite nanocrystals can be tuned by varying the ligand concentration and chain length.^59,60^ The PNCs emitting at 732 nm therefore serve as a proof-of-concept demonstration that recycled MAPbI_3_ powder can be repurposed as a luminescent nanomaterial. This conversion of MHP films into emissive PNCs highlights the versatility of our recycling strategy, while reducing lead-containing waste and providing a potential route to functional materials for next-generation optoelectronic applications.

## 4. Conclusions

In summary, we have developed a green, efficient, and low-cost recycling strategy for MAPbI_3_ thin films using an aqueous solvent system composed of malic acid, β-alanine, and sodium iodide. Combined FTIR, ^1^H NMR, and Raman spectroscopy reveal that the dissolution mechanism relies on the formation of adducts between malic acid and lead species *via* Pb–carboxylate interactions, while the incorporation of NaI is critical to promote iodide-rich lead species in solution and ensure re-precipitation of MAPbI_3_ rather than PbI_2_, upon drying.

Thin films prepared from the recovered MAPbI_3_ were compared with those prepared from fresh perovskite precursor solutions. The phase purity and elemental composition of recycled films show negligible differences from fresh perovskite films, with minor variations in XPS-derived C/N and Pb/I ratios attributable to known measurement artifacts (surface contamination and X-ray-induced reduction under vacuum) rather than intrinsic compositional differences. Films prepared from recycled MAPbI_3_ exhibited slightly larger, more uniform grain sizes and a modestly longer average photoluminescence lifetime (18.7 ns *vs.* 16.8 ns) compared to fresh precursor films, the latter arising primarily from a redistribution in the relative amplitudes of the decay components rather than a change in the underlying carrier dynamics. These results indicate that performance is largely retained, rather than enhanced, through recycling.

Photoconductor devices prepared from recycled MAPbI_3_ exhibited higher photocurrent and lower dark current compared to those fabricated from fresh MAPbI_3_. To further validate device-relevant performance, full p–i–n photovoltaic devices incorporating recycled MAPbI_3_—comprising a solution-processed MeO-2PACz hole transport layer and a thermally evaporated C_60_/BCP electron transport layer—were fabricated and benchmarked against pristine counterparts. These devices achieved average power conversion efficiencies of 16.5% (recycled) *versus* 17.5% (pristine), retaining >90% of pristine performance, with select recycled devices reaching PCEs up to 18%, comparable to pristine analogues. This demonstrates that the recovered material remains fully compatible with complex, multi-interface device architectures without compromising performance. Additionally, the feasibility of producing high-quality rod-shaped perovskite nanocrystals from recycled powders further expands the application scope of our method, highlighting the versatility of the solvent system beyond thin-film reuse alone.

Overall, our approach offers a sustainable, low-cost, and scalable solution for lead recovery and MAPbI_3_ reuse, demonstrating performance comparable to pristine materials across multiple structural, optoelectronic, and device-level metrics. This establishes a green and efficient route that supports a more circular lifecycle for emerging perovskite optoelectronic markets.

## Conflicts of interest

There are no conflicts to declare.

## Supplementary Material

MH-OLF-D6MH01719C-s001

## Data Availability

The data supporting the findings of this study are available in the supplementary information (SI) accompanying this article. Additional data requests can be made to the corresponding author upon reasonable request. Supplementary information is available. See DOI: https://doi.org/10.1039/d6mh01719c.
