# Supplementary material for: Closing the perovskite loop: aqueous recovery and reuse of MAPbI3 for film, device, and nanocrystal reuse
Source: Mater Horiz. 2026 Oct 21. Online ahead of print. doi: 10.1039/d6mh01719c (PMC13717556; doi:10.1039/d6mh01719c)
Supplement: Supplementary file 1 [file MH-OLF-D6MH01719C-s001.pdf]

Supplementary Information (SI) for Materials Horizons.
This journal is © The Royal Society of Chemistry

Closing the Perovskite Loop: Aqueous Recovery and Reuse of $MAPbI_3$ for Film, Device, and Nanocrystal Reuse

Zhaoyi Du[1], Ding Ding[2], Longren Li[2], Weitao Qian[2],Enny Tran[1,2], Milo S. P. Shaffer[1,2], Artem Bakulin[2], Nicola Gasparini[2] and Martyn A. McLachlan[2*]

[1.] Department of Materials, Molecular Sciences Research Hub, Imperial College London, W12 0BZ

[2.] Department of Chemistry, Molecular Sciences Research Hub, Imperial College London W12 0BZ

Experimental Section

Materials

DL-Malic acid (98%), β-alanine (99%), sodium iodide (NaI, ≥99.5%), hydriodic acid (57 wt. % in H_2O), 2-Propanol (IPA, ≥99.5%), diethyl ether (anhydrous, ≥99.0%) were purchased from Sigma Aldrich. Methylammonium iodide (MAI, >99.99%) was purchased from Great Cell Solar. Lead iodide (PbI_2, 99.99 %) was purchased from TCI. SnO_2 nanoparticle dispersion (15% in H_2O) was purchased from Thermo Scientific Chemicals. MeO-2PACz (>98.0%) was purchased from Tokyo Chemical Industry (TCI). C_{60} (>99.5%) was purchased from Ossila. Bathocuproine (BCP, >99.5%) was purchased from Lumtec. All chemicals were used as received.

Preparation of Aqueous Solvent for perovskite recycling

Malic acid and β-alanine were added to deionized (DI) water at the molar ratio of 0.17:1:100 (Malic acid: β-alanine: H_2O). The solutions were stirred at room temperature for 1-hour to ensure complete dissolution, to which NaI was added at a concentration of 0.8g /mL.

Preparation of perovskite precursor solutions

Conventional precursor for fresh films: 1.4 mmol PbI_2 and 1.4 mmol MAI are added in 1 mL of organic solvent (DMF: DMSO = 4:1) in the glovebox. The solution was stirred at 60°C for half an hour to get fully dissolved.

Recycled precursor based on recycled $MAPbI_3$ powder: 1.4 mmol $MAPbI_3$ powder was added to 1 mL of organic solvent (DMF: DMSO = 4:1) in the glovebox. The solution was stirred at 60 □C for half an hour to get fully dissolved.

Perovskite film deposition and photoconductor fabrication

Cut-glass substrates (1.2×1.2 cm and 2×2 cm) were cleaned by sequential immersion in DI water (Decon 90 surfactant), DI water, acetone, and isopropanol, each for 15 minutes with the aid of ultrasonics. After washing the substrates were dried in a nitrogen flow before finally undergoing 15-minutes of treatment in a UV-ozone cleaner.

To fabricate $MAPbI_3$ photoconductors used in the demonstration of selective dissolution of perovskite in aqueous solution (Figure 1), 1.2×1.2 cm glass substrates and 6-pixel mask were used. 35 μL conventional precursor was spin-coated on cleaned glass at a rotational speed of 4000 rpm for 20 seconds in the glovebox. At the seventh second, 400 μL diethyl ether was deposited as anti-solvent. The films were transferred in hot plate for heat annealing at 100 °C for 30 minutes. The thickness of the resultant films is around 400 nm (Figure 3a). 50 nm Ag was thermally evaporated (base pressure 5×10^{-6} mbar) through a shadow mask, creating six Ag electrodes per substrate with the size of 0.3 × 0.45 cm.

To compare the properties of recycled and fresh $MAPbI_3$ films, 2×2 cm glass substrates and masks were used. 60 μL conventional precursor (for fresh films) or 60 μL recycled precursor (for recycled films) was spin-coated on cleaned glass at a rotational speed of 4000 rpm for 20 seconds in the glovebox. At the seventh second, 500 μL diethyl ether was deposited as anti-solvent. The films were transferred in hot plate for heat annealing at 100 °C for 30 minutes. 50 nm Ag was thermally evaporated (base pressure 5×10^{-6} mbar) through a shadow mask with complex geometries, creating 30 devices per-substrate, each with an area of 0.0004 cm^2.

Recycling of $MAPbI_3$ films and devices

Our photoconductor devices were fully immersed in 5 mL of aqueous solvent at 80 °C and stirred until the perovskite is dissolved and the glass substrate underneath is exposed. Following dissolution of the perovskite – the removal of the perovskite from the substate could be detected visibly – the substrate was removed and the solution cooled to around 40 °C, resulting in the formation of a precipitate (black α-phase $MAPbI_3$). The powder was subsequently centrifuges and washed using **h**ydriodic acid/ IPA and centrifugates again, the wash-centrifugate process was repeated 4-times. The subsequent powders were collected and dried in air using a low-temperature oven (60 °C) overnight. The obtained dry $MAPbI_3$ powder was transferred and stored inside the glovebox for further uses.

Assessment of recovery yield

To evaluate the recovery yield of the fresh and reused aqueous solvent, $MAPbI_3$ with certain mass (weighted as MAI and PbI_2 with equal moles) was added into the aqueous solvent at 80 °C and then cooled to 40 °C. The precipitates were separated by centrifugation, and the supernatant was reused for new round of recovery. The obtained precipitates were washed and dried. The mass of the recovered precipitates was weighted and used to evaluate the recovery yield.

Assessment of selectivity of aqueous solvent

Selectivity towards NiOx: Nickel acetate solution (H_2O as the solvent) was spin-coated onto the ITO glass and annealed at 300°C to obtain a thin layer of NiOx. The photo and XRD pattern of the NiOx layer were taken. The NiOx layer was then immersed in aqueous solvent for 3 minutes and washed with DI water. The photo and XRD pattern of the NiOx layer after immersion were taken again to compare.

Selectivity towards SnO_2: SnO_2 solution (H_2O, 15% colloidal dispersion) was spin-coated onto the ITO glass and annealed at 100°C to obtain a thin layer of SnO_2. The photo and XRD pattern of the SnO_2 layer were

taken. The SnO_2 layer was then immersed in aqueous solvent for 3 minutes and washed with DI water. The photo and XRD pattern of the SnO_2 layer after immersion were taken again to compare.

Preparation of fresh and recovered $MAPbI_3$ solar cells

MAI and PbI_2 were added in DMF: DMSO (9:1) to prepare the fresh perovskite precursor solution. The prepared $MAPbI_3$ films were subsequently dissolved in the aqueous solvent and precipitated to obtain the recovered $MAPbI_3$ powders, which were re-dissolved in DMF: DMSO (9:1) to make the recycled perovskite precursor solution used for device preparation.

The indium tin oxide (ITO)-coated glass substrates were cleaned by sequential immersion in DI water (Decon 90 surfactant), DI water, acetone, and isopropanol, each for 15 minutes with the aid of ultrasonics. After drying, the substrates were exposed to UV-ozone for 30 minutes and subsequently transferred into a nitrogen-filled glovebox for spin-coating. 30 μL of MeO-2PACz in ethanol (1 mmol/mL) was spin-coated onto the ITO at 3000 rpm for 30 s (acceleration of 1500 rpm s^{-1}). The resulting film was heat-annealed on a hotplate at 100 °C for 10 minutes. Fresh and recycled $MAPbI_3$ precursor solutions were spin-coated at 4000 rpm for 20 seconds. After 7 seconds, 400 ul of diethyl ether was dripped on top. The film was heat annealed for 2 minutes on a hot plate at 60 °C, then transferred to a hot plate at 100 °C for 60 minutes. The device stack was completed by sequential thermal evaporation of 30 nm C_{60}, 8 nm bathocuproine (BCP) and 100nm Ag at a base pressure of 5×10^{-6} mbar. These layers served as the electron transport layer, hole-blocking layer and top electrode, respectively. BCP and Ag were deposited through a shadow mask, defining an active pixel area of 0.045 cm^2.

Synthesis of $MAPbI_3$ nanocrystals from recycled $MAPbI_3$ powders

The synthesis method is modified based on a previous work.[1] Specifically, 0.1 mmol (62 mg) recycled $MAPbI_3$ powder was dissolved in 1 mL of DMF containing 100 μL of oleyamine and 150 μL of oleic acid. 15 μL of this precursor solution was then dropped into 3 mL of chloroform followed by vigorous, room temperature stirring for 10-seconds, during which time a distinct colour change is seen and when illuminated by a UV-source an intense red emission is seen. The solution was centrifuged at 5000 rpm for 10-minutes with any precipitates discarded to obtain the nanocrystal dispersion.

Materials characterisation

Fourier transform infrared spectroscopy (FTIR) was conducted using an Agilent Cary 630 FTIR spectrometer equipped with an attenuated total reflectance (ATR) sampler.

Proton nuclear magnetic resonance (^1H NMR) was conducted on Bruker AV-400 spectrometer. D_2O is used as the solvent.

Raman spectroscopy was conducted using a Renishaw inVia confocal Raman microscope equipped with a 633 nm excitation wavelength laser source (maximum power of 20 mW), diffraction grating of 1800 lines mm^{-1}, and 50x objective lens to focus the laser on the sample with an average laser spot diameter of approximately 1.6 μm. Prior to the measurements, a calibration step was performed using an integrated silicon (Si) wafer and the diffraction grating position was offset to account for drift with respect to the characteristic

Si Raman band at 520 cm^{-1}. The laser exposure time was 30 s under a laser intensity of 0.2 mW. The samples were measured across the Raman shift range 50-550 cm^{-1}. To prepare the samples, $MAPbI_3$ films were dissolved in aqueous solutions of malic acid/β-alanine/NaI/H_2O and malic acid/β-alanine/H_2O, respectively. The solutions were dried overnight to remove the water and obtain viscous $MAPbI_3$-malic acid adducts, which were then loaded on glass substrates for Raman measurement. The sample of $MAPbI_3$ thin film (400 nm) was deposited on a glass substrate for the measurement.

UV-vis absorption spectra were collected using an Agilent Cary 60 in absorption mode.

X-ray diffraction (XRD) patterns were acquired using a PANalytical MPD instrument operated at 40kV and 40mA.

X-ray photoelectron spectroscopy (XPS) was performed using a Thermo Fisher K-Alpha$^+$ equipped with a monochromate Al K_a micro-focused X-ray source. (Al Ka = 1486.6 eV). The analysis chamber was under the vacuum level of 1×10^{-7} with flood gun on. Photoelectrons were analysed using a 180-degree double-focusing hemispherical analyser equipped with a 128-channel detector. Survey spectra were acquired at a pass energy of 200 eV, while the core-level spectra were acquired at a pass energy of 35 eV.

Time-correlated single photon counting (TCSPC) was conducted with the HORIBA DeltaFlex™ system (detector: PPD-900, Horiba scientific). A 467 nm laser with < 200 ps pulse duration (NanoLED, Horiba scientific) was used for excitation (repetition rate: 1 MHz). The measurement of the film was at 770 nm.

Scanning electron microscopy (SEM) images were obtained using a Zeiss Auriga Cross Beam FIB-SEM, at an accelerating voltage of 5kV and working distance at 5 mm. Prior to imaging a thin (< 10 nm) layer of Au was sputtered onto the film surface to facilitate imaging.

Steady -state photoluminescence (PL) spectra were acquired by Agilent Cary Eclipse Fluorescence Spectrophotometer. The wavelength of excitation for $MAPbI_3$ films and nanocrystals was 465 nm.

Photoluminescence (PL) mapping was performed using a custom-built optical microscope setup. Specifically, a continuous-wave 405 nm laser diode (Thorlabs) was focused onto the sample surface through a 10× objective lens (Mitutoyo, NA = 0.25), delivering approximately 10 μW of average power at the sample plane. The emitted PL was collected by the same objective, collimated, and subsequently focused by a lens (f = 3 mm) onto an avalanche photodiode (APD430A, Thorlabs Inc.) for photon counting.

Femtosecond pump–probe transient absorption (TAS) measurements were performed on a commercial broadband spectrometer (Helios, Spectra-Physics/Newport). Fundamental pulses at 800 nm (∼100 fs, 1 kHz) were provided by a Ti:sapphire regenerative amplifier (Solstice, Spectra-Physics). A fraction of the 800 nm output was sent to an optical parametric amplifier (TOPAS Prime, Spectra-Physics) combined with a frequency-mixing module (Light Conversion) to generate tunable visible pump pulses in 400 nm. Pump modulation was achieved with a mechanical chopper operating at 500 Hz.

Photoconductor characterisation

Current-voltage (I-V) characteristics were measured by a Keithley 4200 Source-Measure unit (scan step 0.2V) equipped with a Remote PreAmp (Model 4200-PA) to extend the resolution to 0.1 fA. A 530 nm LED (Thorlabs M530L4 LED, 370 mW, 1000 mA) was used as the light source. The light intensity applied in the measurement was increased from 1.59 mW cm^{-2} to 25.39 mW cm^{-2}.

Table S1. Comparison of existing literature reports of perovskite recycling with our work.

Year	Perovskite composition	Material recovered	Solvent	Mechanism	Recovery efficiency	Post recycling performance	Ref
2016	$MAPbI_3$	$MAPbI_3$ (indirect)	DMF, GBL, DMSO	Solvent – pero. interaction	N/A	≈ 100%	https://doi.org/10.1038/ncomms11735
2021	$Cs_{0.1}FA_{0.9}PbI_3$	PbI_2	DMF + Carboxylic acid cation-exchange resin+ NaI	Solvent – pero. interaction + Pb^{2+} formation + PbI_2 precipitation	99.2%	97%	https://doi.org/10.1038/s41467-021-26121-1
2023	$FA_{0.83}Cs_{0.17}PbI_3$	PbI_2	H_2O	FAI /CsI soluble in H_2O; PbI_2 precipitation	92.6 -100%	N/A	https://doi.org/10.1016/j.jhazmat.2023.130829
2023	$MAPbI_3$	PbI_2	H_2O	PbI_2 precipitation	100%	N/A	https://doi.org/10.1016/j.jhazmat.2023.130829
2024	$MAPbI_3$	$MAPbI_3$	GBL/ethyl acetate antisolvent	Solvent – pero. Interaction. Ethyl-acetate precipitation of $MAPbI_3$	87%	97%	https://doi.org/10.1039/d4ee01071j
2025	$MAPbI_3$	$MAPbI_3$	H_2O, NaOAc, NaI, H_3PO_2	Forming Pb^{2+} - acetate complex	99%	99%	https://doi.org/10.1038/s41586-024-08408-7
2025	$FAPbI_3$	PbI_2	H_2O	H_2O dissolution of $FAPbI_3$ at low concentration; EtOH precipitation of PbI_2	99%	98.4%	https://doi.org/10.1039/d4el00004h
2026	$MAPbI_3$	$MAPbI_3$	H_2O, malic acid, β-alanine, NaI	Forming $PbI_2(\mathit{Sol})_n$ adducts	24% (fresh) 82% (1st reuse) 85% (2nd reuse)	94%	Current work

Table S2. Position of fitted Raman peaks for $MAPbI_3$ film, $MAPbI_3$ dissolved in aqueous solvents with/without NaI, under 633 nm excitation.

**$MAPbI_3$ film**	**$MAPbI_3$ in aqueous solvent (Without NaI)**	**$MAPbI_3$ in aqueous solvent (With NaI)**
Peak position (cm^{-1})	Peak position (cm^{-1})	Peak position (cm^{-1})
84	61.3	96.3
131.2	86.5	110.9
224	106.1	136.5
264	-	158.1
320	-	-
381	-	-
445	-	-

Table S3. Comparison of the toxicological hazards associated with organic and aqueous solvent used in perovskite recovery.

Medium	Recovery materials	Principal health hazards	Relevant exposure routes	Overall risk level
DMF [a]	Perovskite	Skin absorption; Cause hepatotoxicity following acute or repeated exposure; Reproductive toxicant; Cause serious eye and respiratory irritation.	Dermal contact, inhalation, and ingestion	High
DMSO [b]	Perovskite	Cause mild skin and eye irritation; Enhance the dermal absorption of dissolved substances and contaminants; Exposure to high concentrations may produce systemic effects, including reduced consciousness.	Dermal contact, ingestion, and inhalation of aerosols	Moderate - High
GBL [c]	Perovskite	Cause severe eye damage and skin absorption; Following ingestion, it is rapidly converted to γ-hydroxybutyrate and may induce pronounced central nervous system depression, respiratory impairment, convulsions, and loss of consciousness.	Ingestion, dermal contact, and inhalation	High, particularly following ingestion
H_2O	PbI_2	Negligible intrinsic chemical toxicity	N/A	Very low
Present work [d]	Perovskite	Malic acid and β-Alanine generally present low acute toxicity at low concentration; Prolonged iodide exposure may affect thyroid function.	Dermal and ocular contact, ingestion	Low - moderate

[a.] International Labour Organization and World Health Organization. *International Chemical Safety Card 0457: N,N-Dimethylformamide*.

[b.] International Labour Organization and World Health Organization. *International Chemical Safety Card 0459: Dimethyl Sulfoxide*.

[c.] International Labour Organization and World Health Organization. *International Chemical Safety Card 1020: γ-Butyrolactone*.

[d.] DL-Malic acid, Safety Data Sheet, ThermoFisher, 18/12/2025;

β-Alanine, Safety Data Sheet, ThermoFisher, 21/12/2025;

Leung, A. M.; Braverman, L. E. Consequences of excess iodine. Nature Reviews Endocrinology 2014, 10, 136–142.

Table S4. Calculated A_1, t_1, A_2, t_2, t_{ave}, k_r and k_{nr} of films (on glass) made with fresh perovskite precursors (fresh film) and recycled perovskite precursors (recycled film).

	**Fresh film**	**Recycled film**
A_1	0.780317472	0.719535783
t_1 (ns)	8.69096 ± 0.07988	9.33904 ± 0.08965
A_2	0.219682528	0.280464217
t_2 (ns)	45.47283 ± 0.2769	42.65508 ± 0.2254
t_{ave} (ns)	16.76992339	18.68366538
Error (ns)	± 0.123	± 0.128

Biexponential function is used for fitting TRPL data as:

$$A(t) = A_1 \exp\left(\frac{-t}{\tau_1}\right) + A_2 \exp\left(\frac{-t}{\tau_2}\right) \quad \text{(S1)}$$

where A_1 and A_2 are the relative ratio of ratios of the two recombination modes, τ_1 and τ_2 are PL lifetime of the charge carriers.

(a) (b) (c)

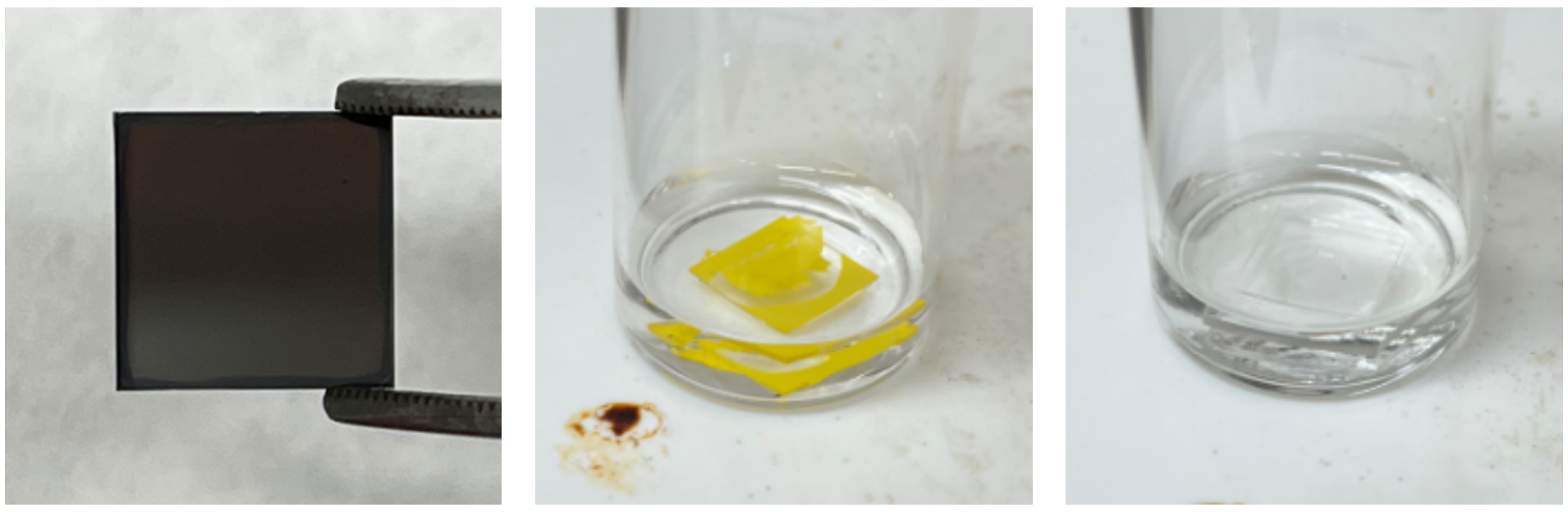

Figure S1. Photographs of $MAPbI_3$ films deposited on glass substrates. (a) Freshly prepared film. (b) Film immersed in water; the observed colour change indicates degradation of $MAPbI_3$ to PbI_2. (c) Film immersed in an aqueous solution containing malic acid and β-alanine.

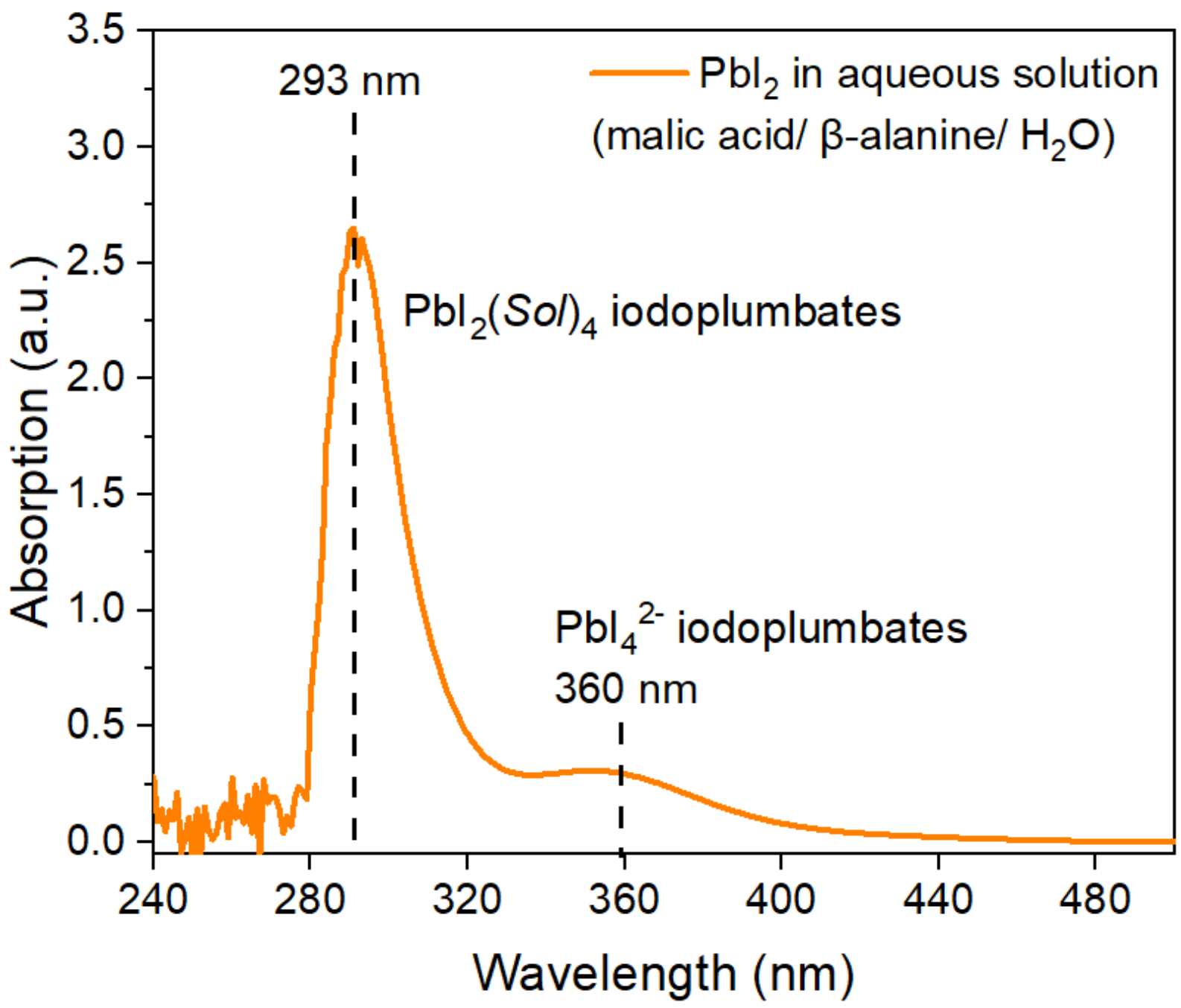

Figure S2. UV-vis absorption spectra of the PbI_2 aqueous solution (malic acid/ β-alanine/ H_2O), confirming adduct formation and stabilization of PbI_2. *Sol* refers to solvent.

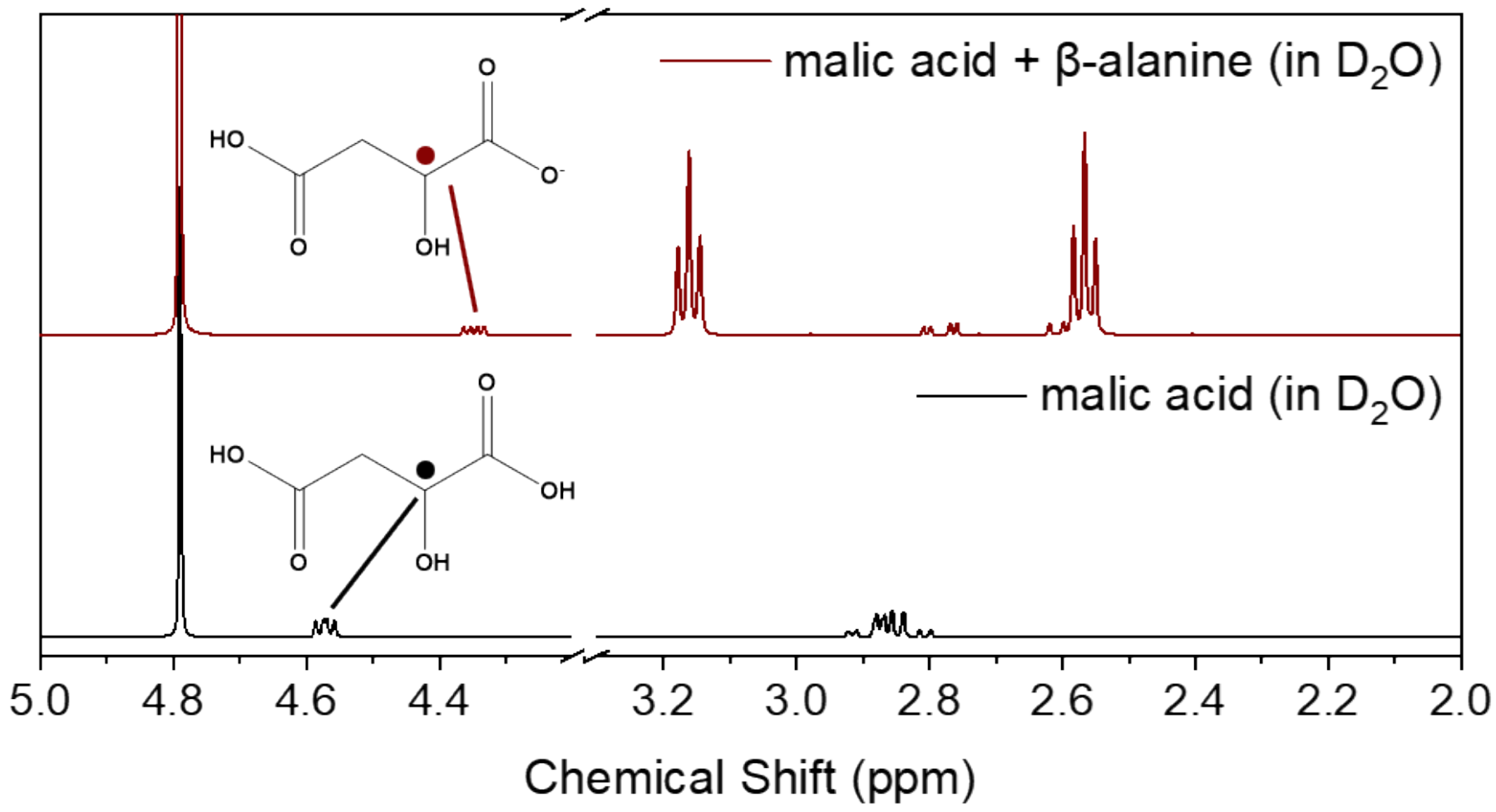

Figure S3. ^{1}H NMR of DL-malic acid, DL-malic acid and β-alanine in D_2O.

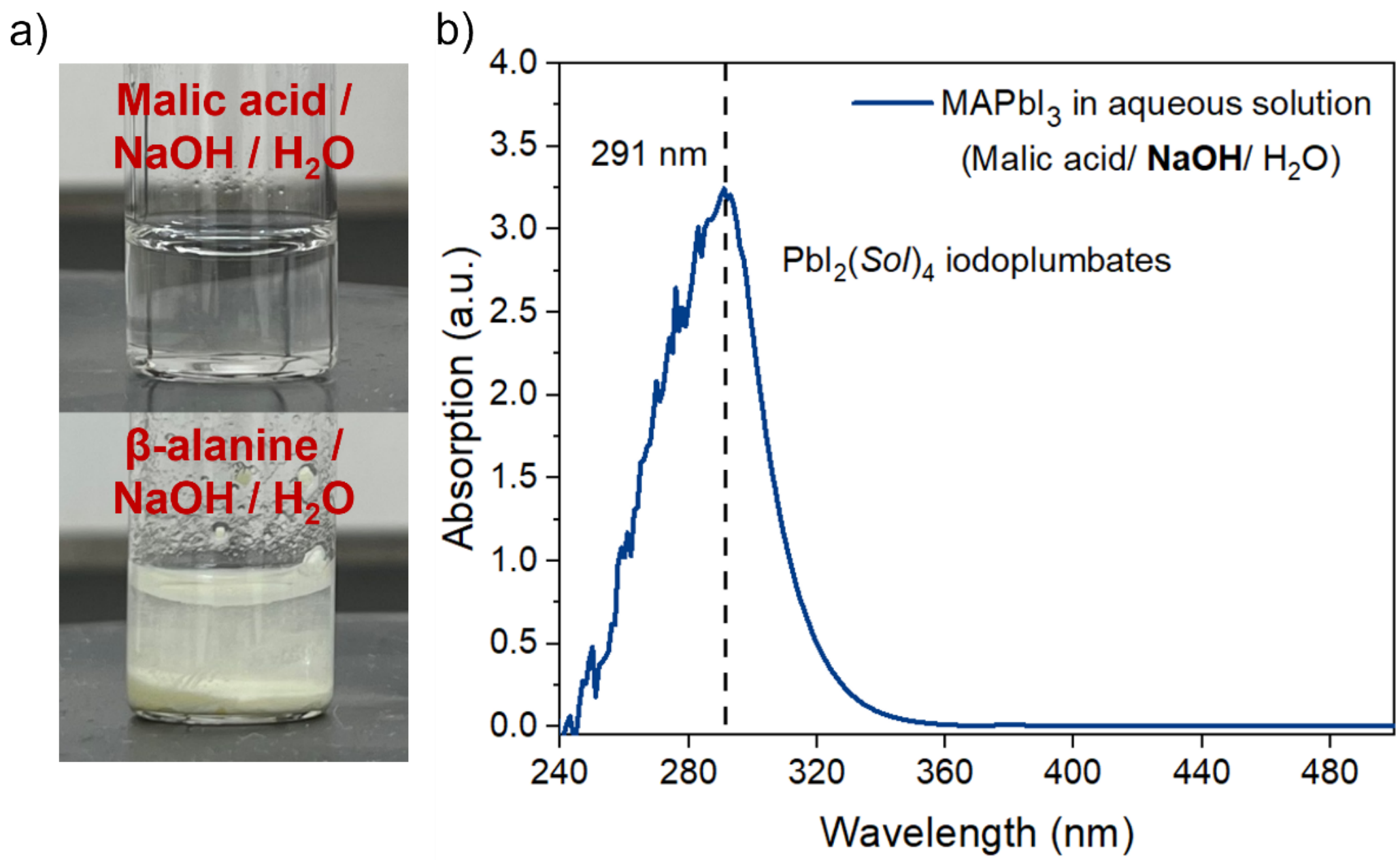

Figure S4. (a) Solubility of $MAPbI_3$ in two aqueous solvent systems: malic acid/NaOH/H_2O and β-alanine/NaOH/H_2O. (b) UV–visible absorption spectra of $MAPbI_3$ dissolved in the malic acid/NaOH/H_2O solvent system. *Sol* refers to solvent.

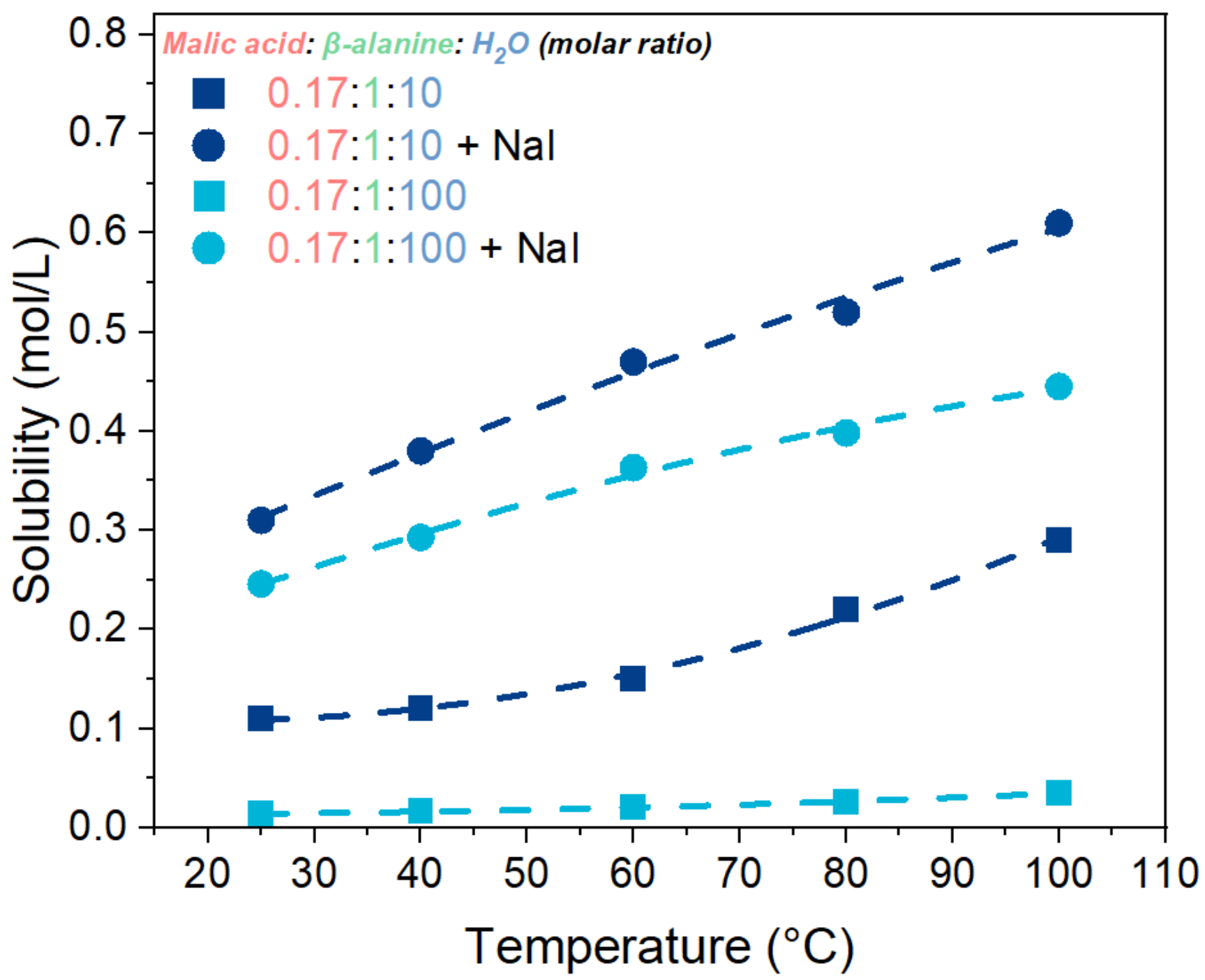

Figure S5. Solubility of $MAPbI_3$ powder in aqueous solutions containing different concentrations of malic acid (MA) and β-alanine (βA), with and without the addition of NaI.

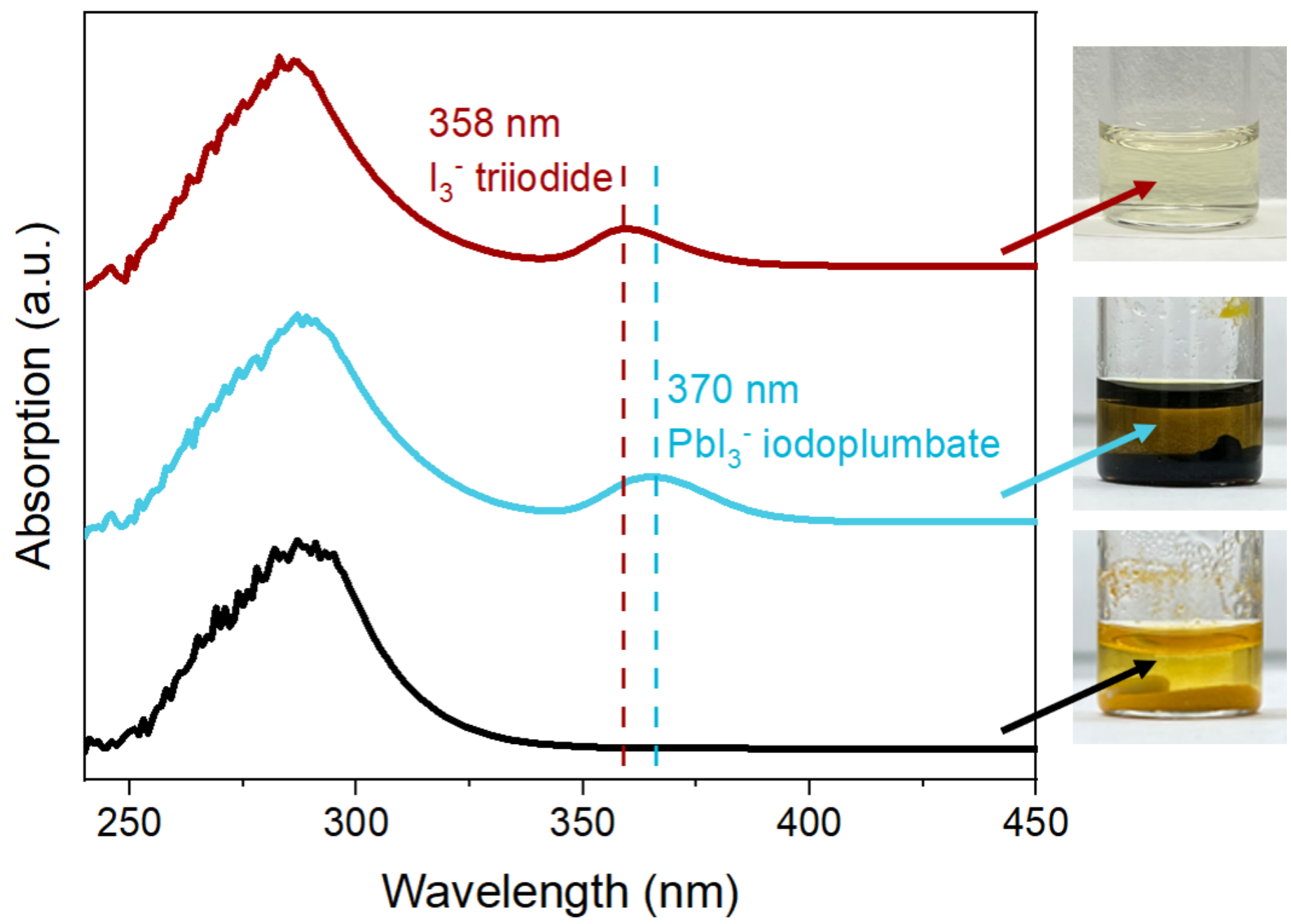

Figure S6. UV–visible (UV-Vis) absorption spectra of the supernatants obtained from $MAPbI_3$ dissolved in malic acid/β-alanine/H_2O (black) and malic acid/β-alanine/NaI/H_2O (blue), together with the spectrum of the corresponding malic acid/β-alanine/NaI/H_2O solvent without $MAPbI_3$ (red). Photographs of the respective solutions are shown to the right.

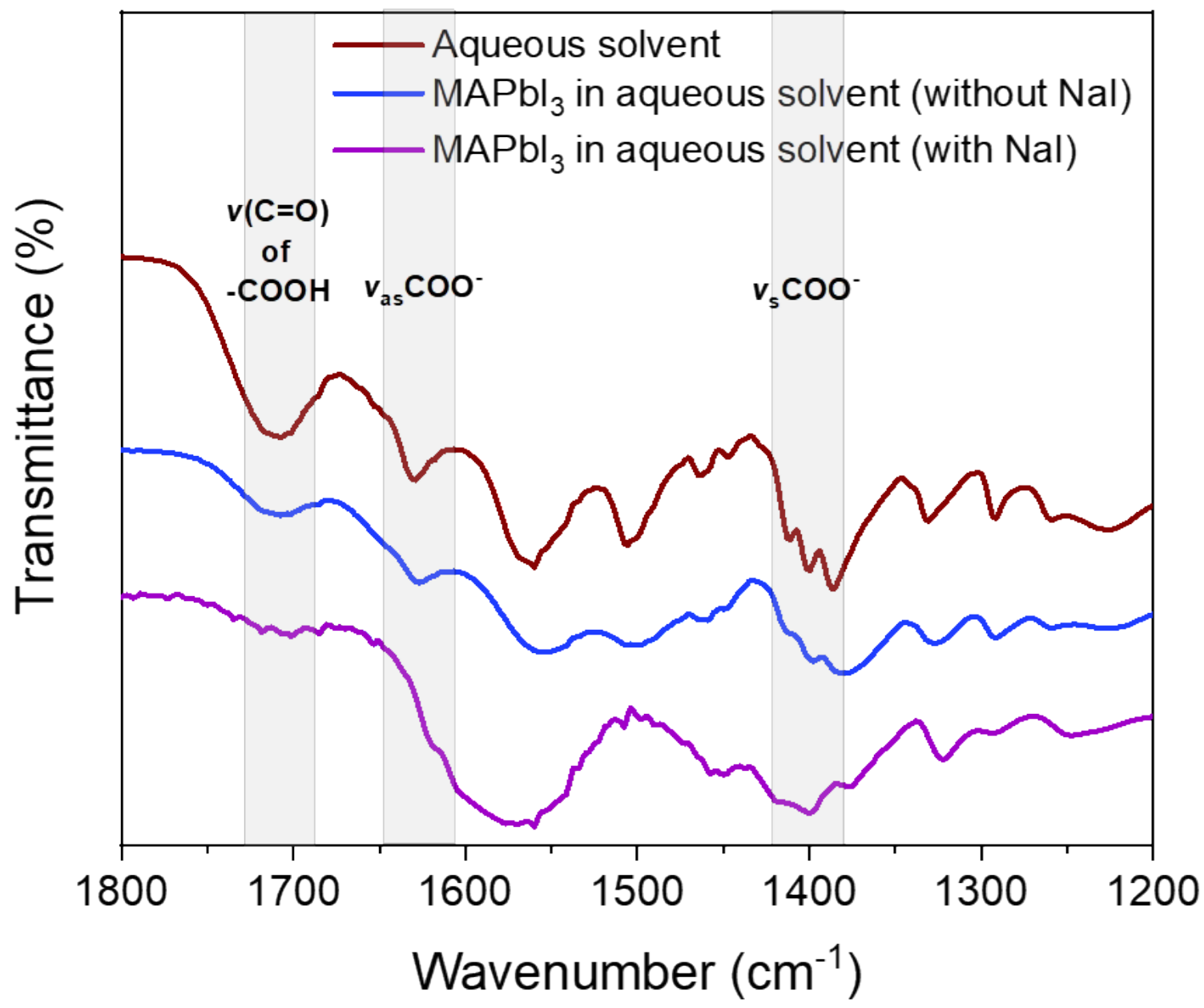

Figure S7. FTIR spectra of aqueous solvent, $MAPbI_3$ dissolved in aqueous solvent and $MAPbI_3$ dissolved in aqueous solvent with addition of NaI.

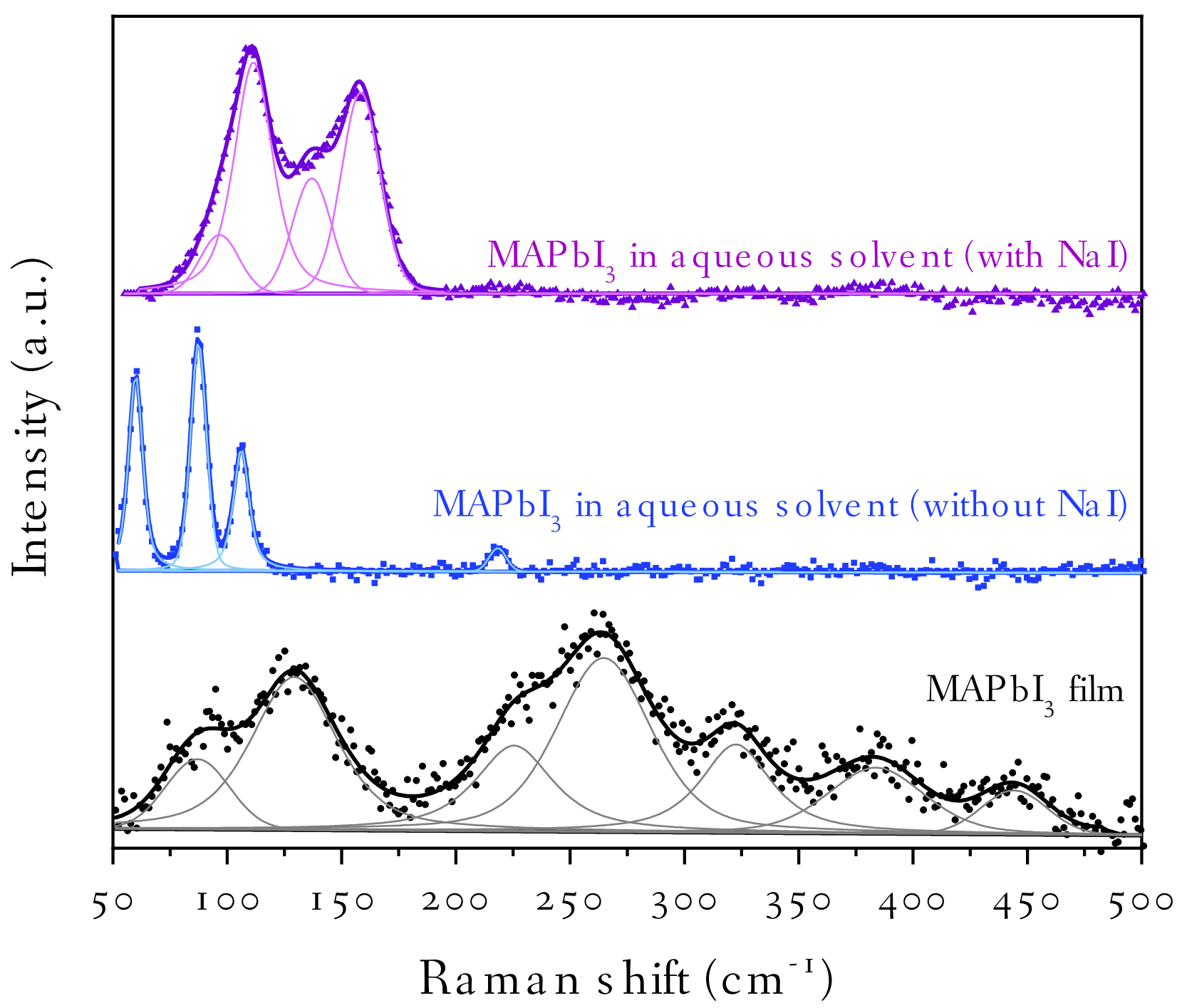

Figure S8. Raman spectra of $MAPbI_3$ film, $MAPbI_3$ dissolved in aqueous solvents with/ without NaI (633 nm excitation)

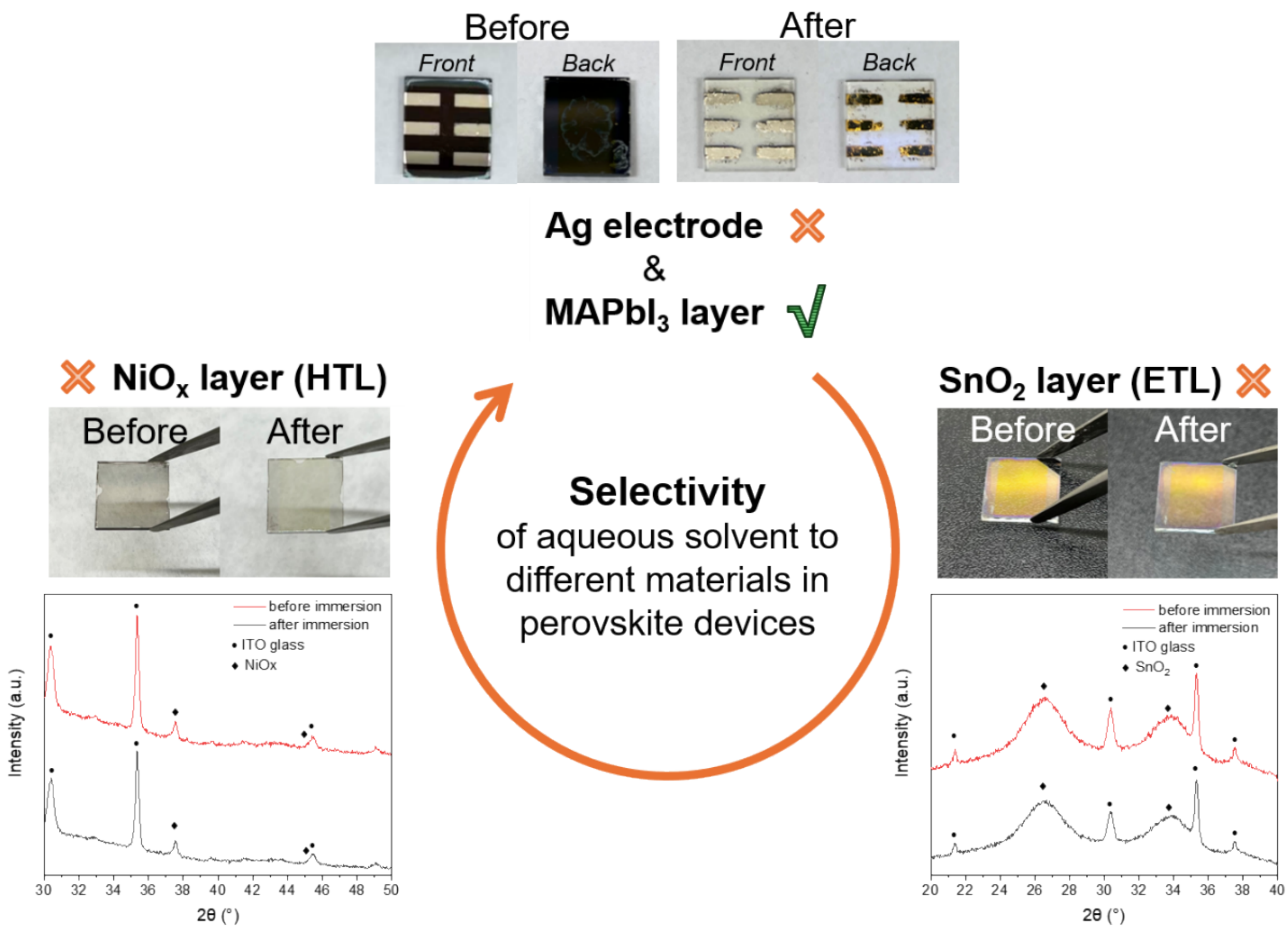

Figure S9. Selectivity of the aqueous solvent towards materials commonly encountered in perovskite devices. Photographs show each material before and after 3-minute immersion in the aqueous solvent. XRD patterns show NiOₓ and SnO_2 films deposited on indium tin oxide (ITO)-coated glass before and after the same - minute immersion. Materials soluble in the aqueous solvent are marked with a check mark (□), while insoluble materials are marked with a cross (×).

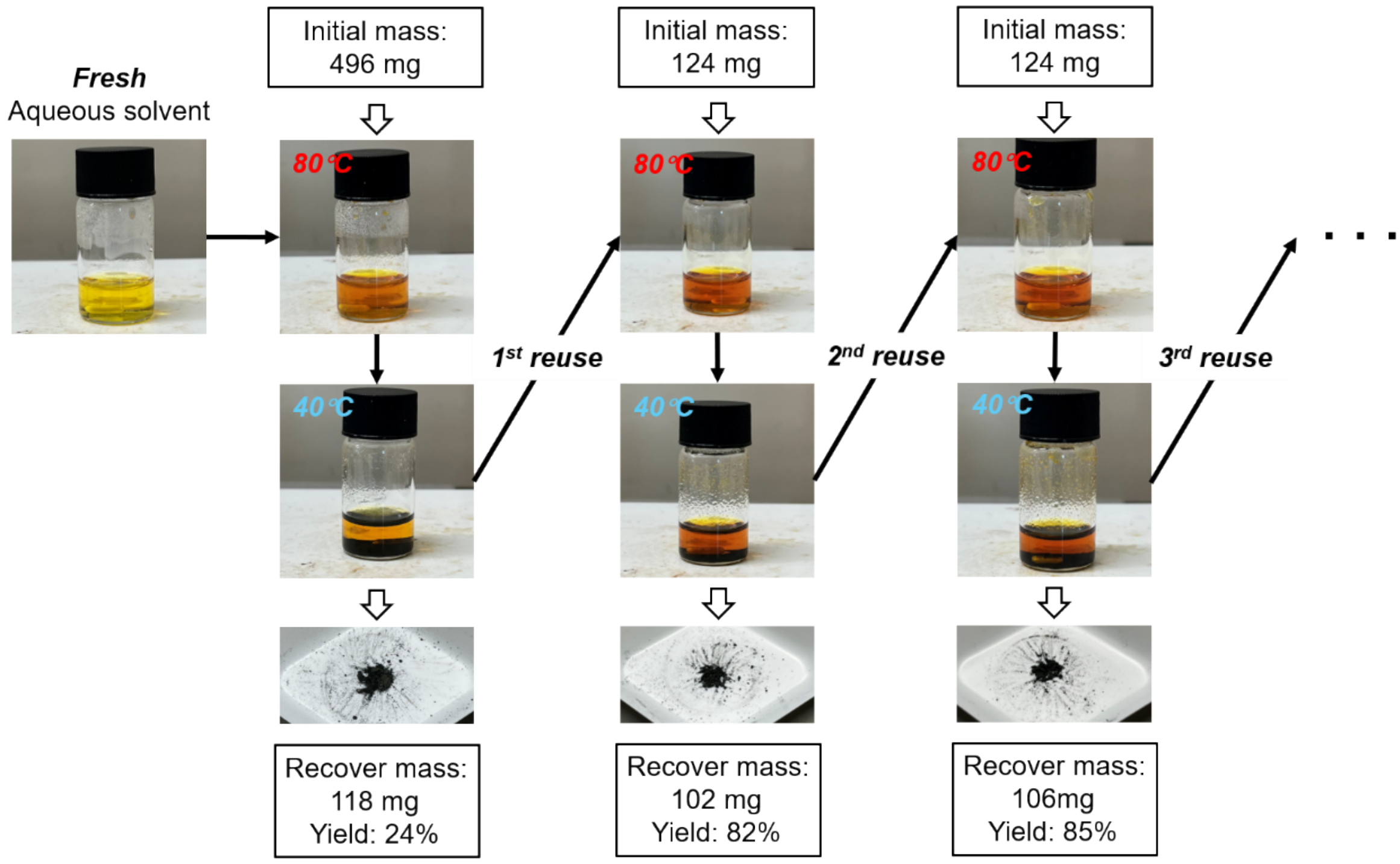

Figure S10. Recovery yield of fresh and reused aqueous solvent. Note , MAI and PbI_2 were mixed to prepare fresh $MAPbI_3$ at each stage such that their mass was known to ensure the yield could be quantified.

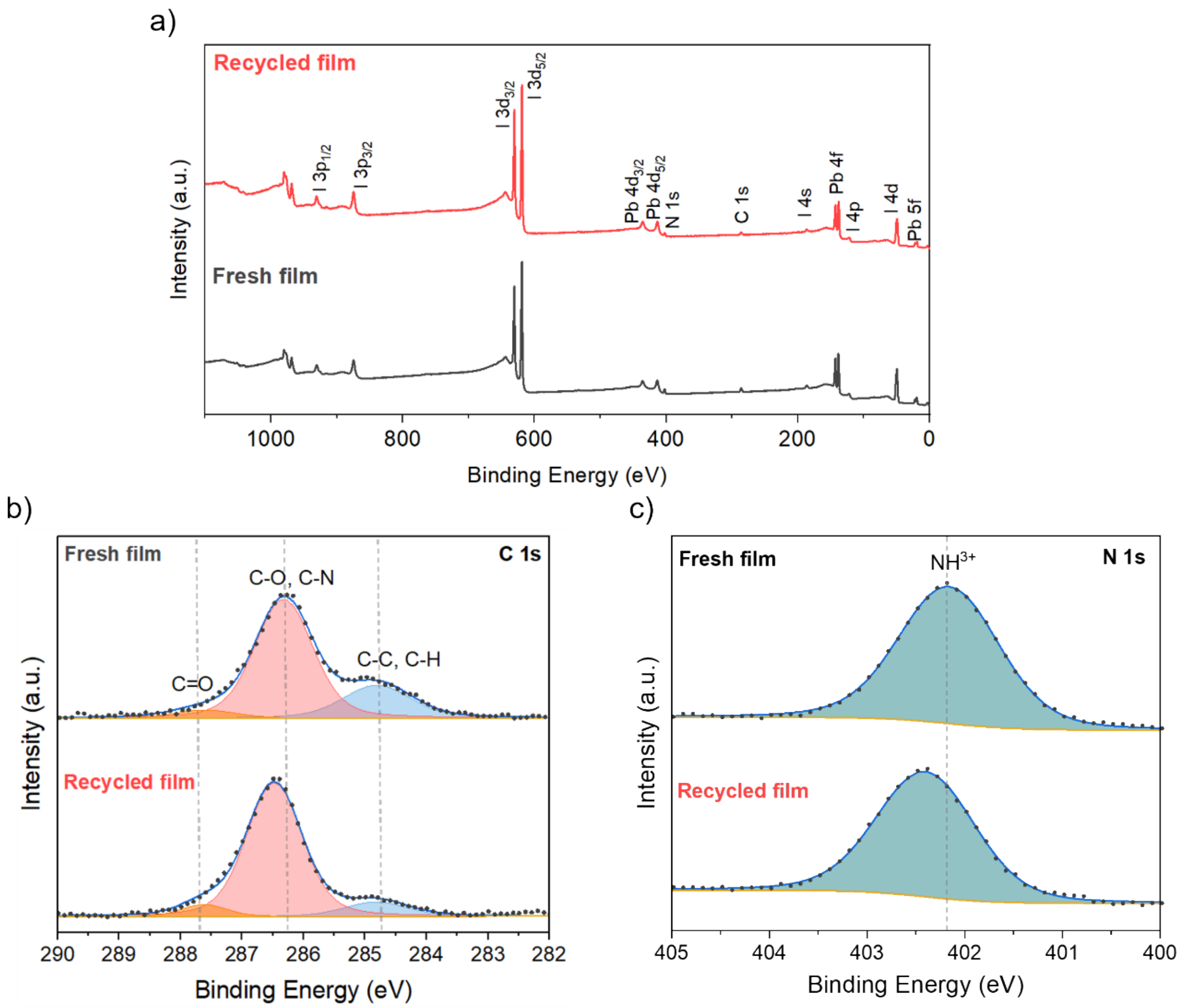

Figure S11. (a) XPS survey spectra and (b) high-resolution C 1s and **(c)** N 1s spectra spectra of $MAPbI_3$ films prepared from fresh precursor and recycled $MAPbI_3$.

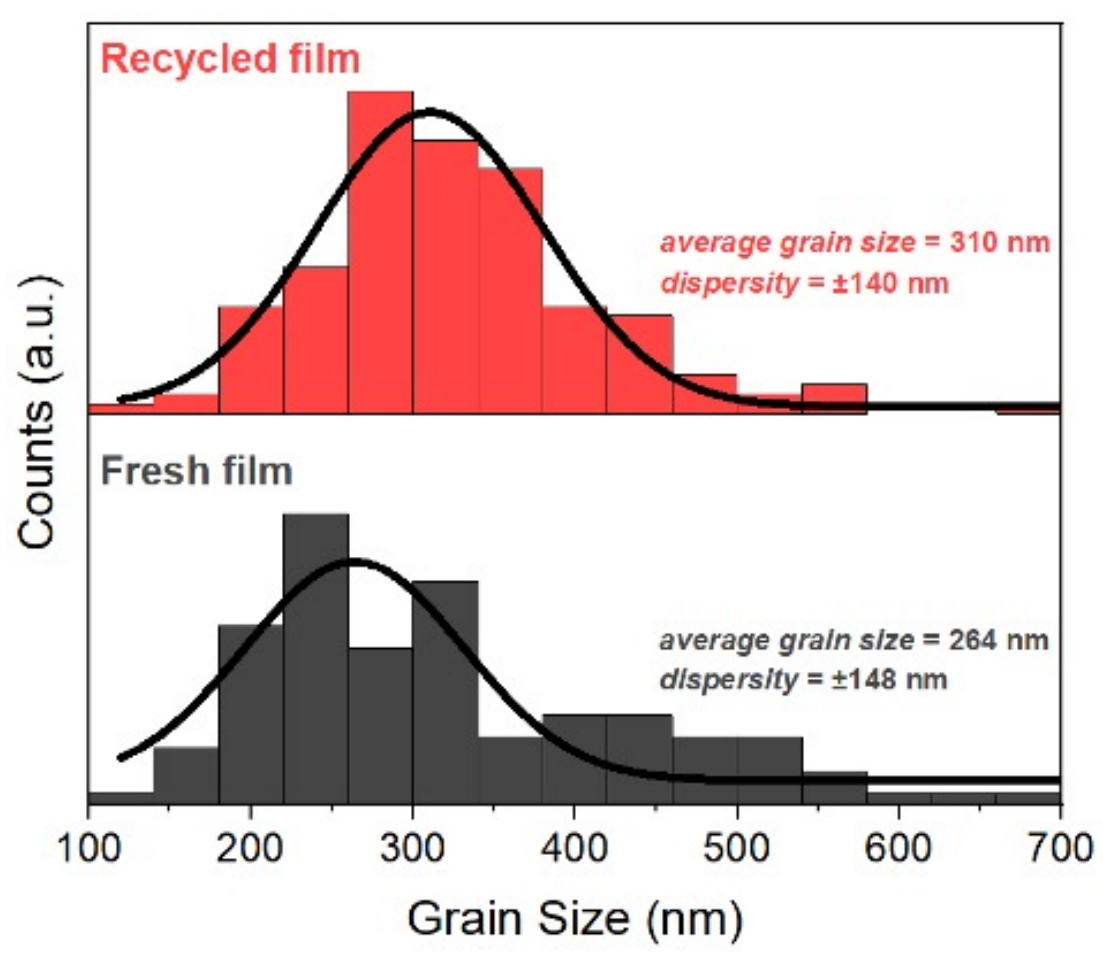

Figure S12. Grain-size distributions of $MAPbI_3$ films prepared from fresh precursor and recycled $MAPbI_3$.

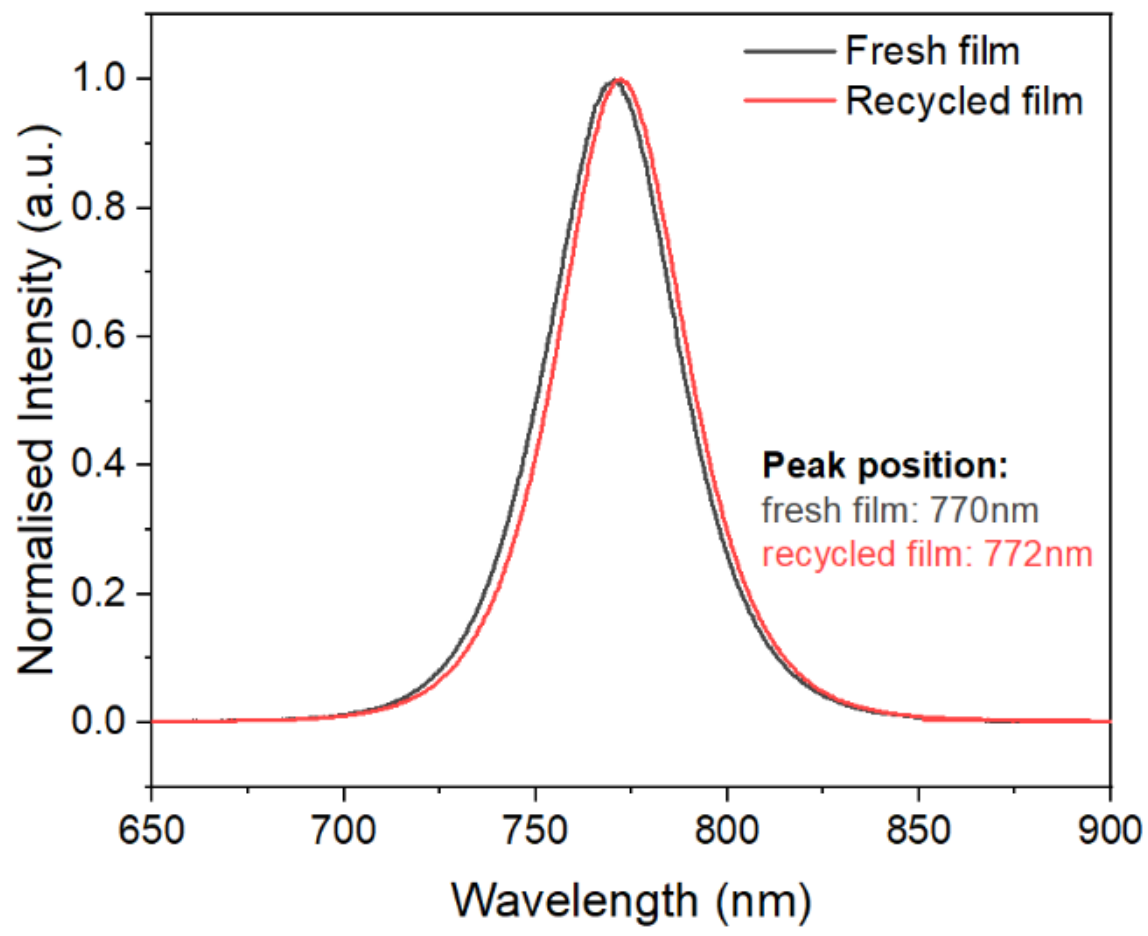

Figure S13. Steady-state photoluminescence spectra of recycled perovskite films and fresh perovskite films.

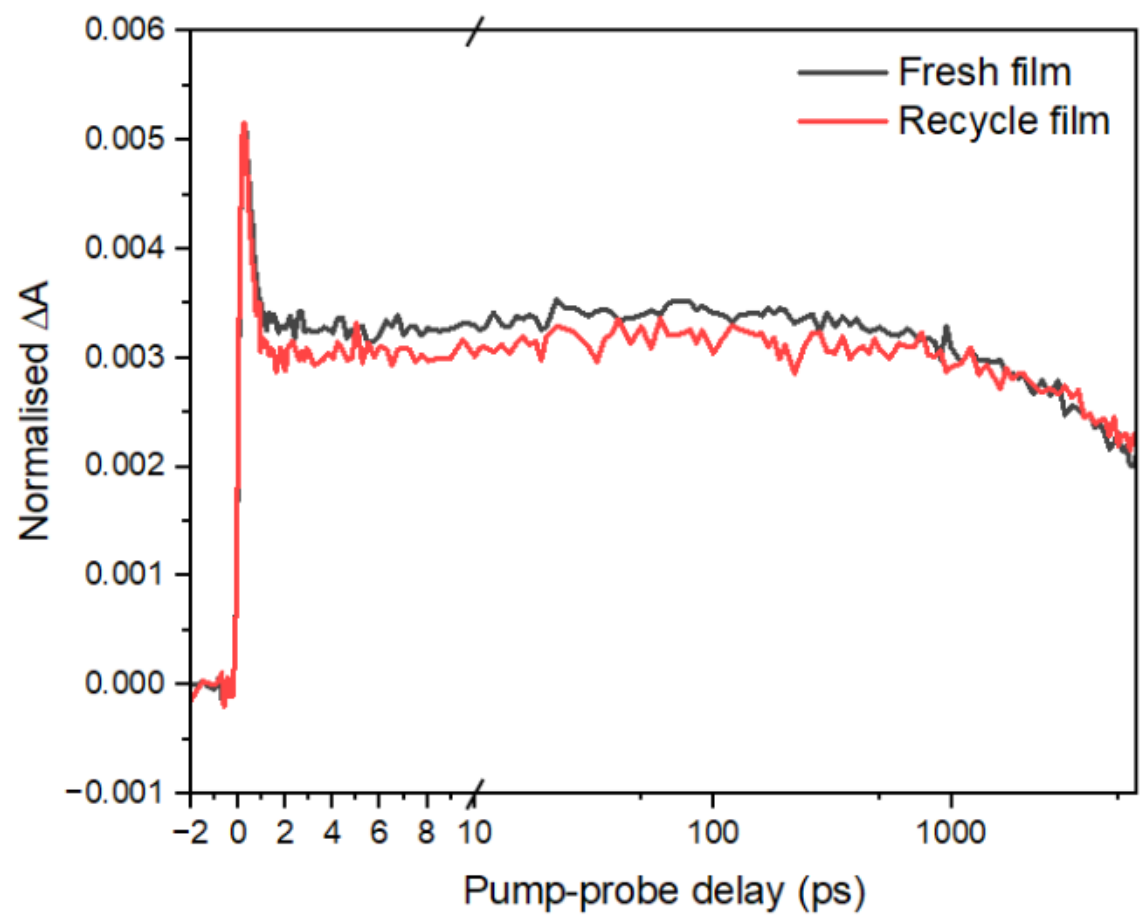

Figure S14. Normalised band-edge bleach kinetics extracted at 750 nm for the fresh and recycled $MAPbI_3$ films following 400 nm excitation at a pump fluence of 0.4 μJ cm^{-2}.

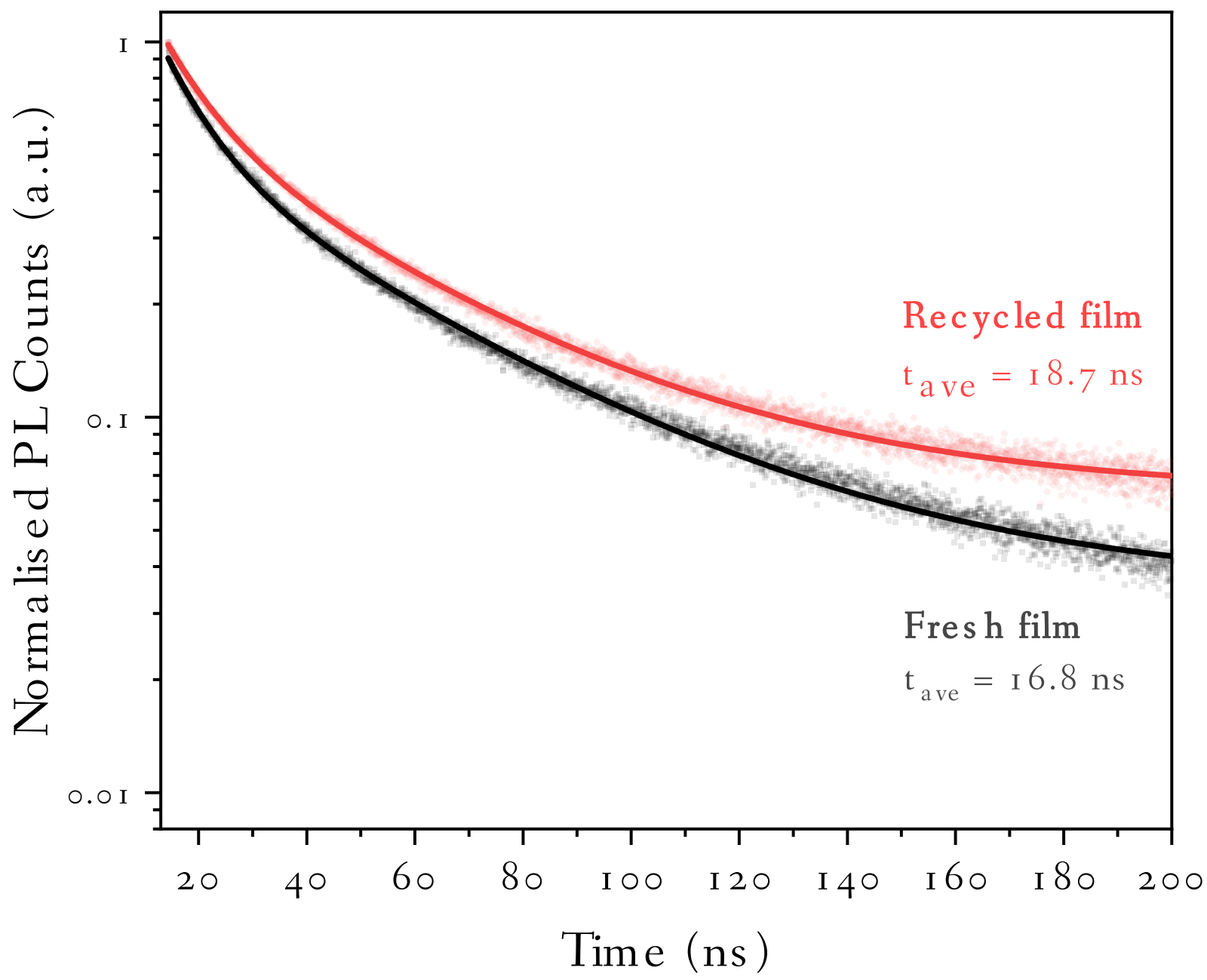

Figure S15. Time-correlated single-photon counting (TCSPC) measurements of $MAPbI_3$ films prepared from fresh precursor (fresh film) and recycled $MAPbI_3$ (recycled film).

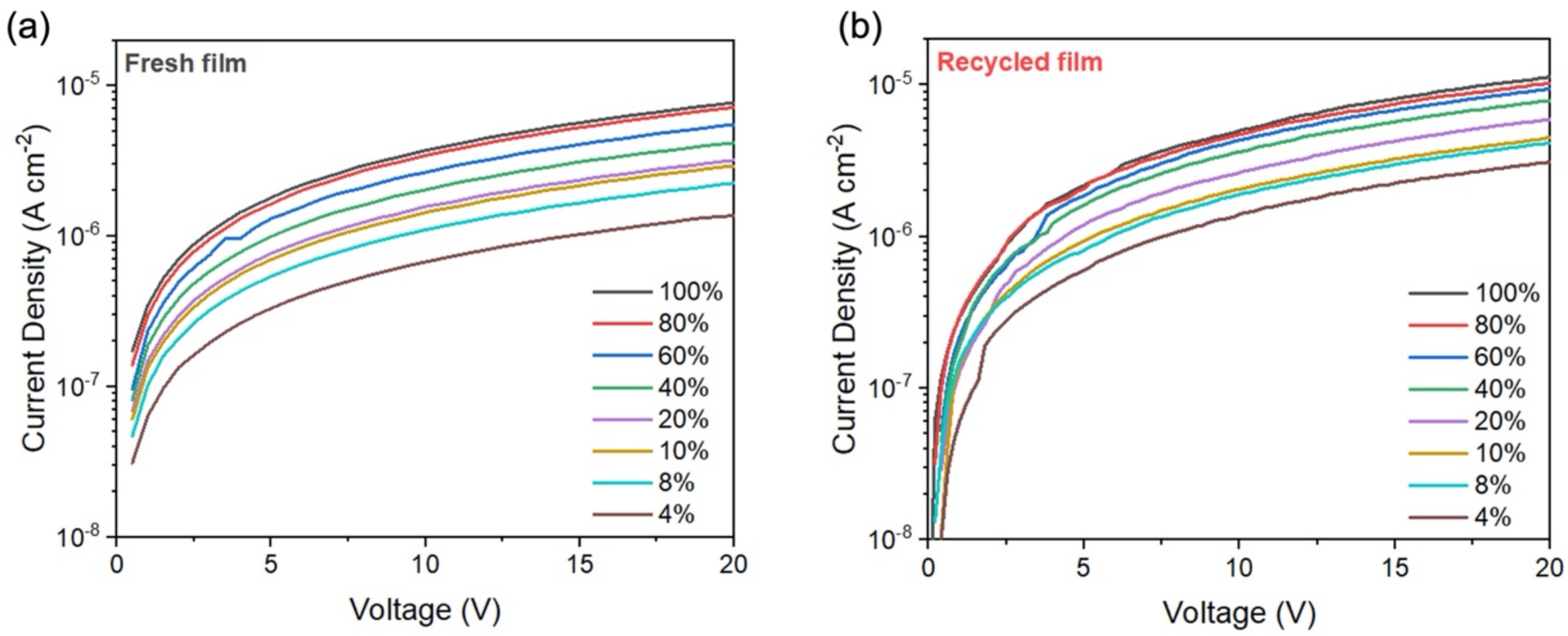

Figure S16. Current density measured for photoconductors fabricated from fresh and recycled $MAPbI_3$ films under varying incident light intensities and applied biases.

References

1 I. Levchuk, P. Herre, M. Brandl, A. Osvet, R. Hock, W. Peukert, P. Schweizer, E. Spiecker, M. Batentschuk and C. J. Brabec, Ligand-assisted thickness tailoring of highly luminescent colloidal CH

$_3NH_3PbX_3$ (X = Br and I) perovskite nanoplatelets, *Chemical Communications*, 2017, 1, **53**, 244–247.
